# Supplementary material for: Fitting dynamic measles models to subnational case notification data from Ethiopia: Methodological challenges and key considerations
Source: PLoS Comput Biol. 2025 Apr 16;21(4):e1012922. doi: 10.1371/journal.pcbi.1012922 (PMC12002527; doi:10.1371/journal.pcbi.1012922)
Supplement: S1 Text — (DOCX) [file pcbi.1012922.s001.docx]

S1 Text.

#

# **Section 1. First- and second-dose coverage of measles-containing vaccines**

*Routine immunization*

Estimates of routine first-dose measles containing vaccine (MCV1) coverage were obtained using methods similar to those described previously^1^. These models leverage household-based survey data, a suite of geospatial covariates, and model-based geostatistical techniques to predict coverage at the 5-x-5 km level. Updated estimates include age-specificity via a space-time-age error term and an age-specific Gaussian process. To account for data aggregated across multiple age groups, we modeled the number of vaccinated children ($C_{i}$) among a sample ($N_{i}$) for a given observation $i$ using the following equations:

$$C_{i}\sim\mathrm{Binomial}\left( N_{i},p_{i} \right)$$

$$\mathrm{logit}\left( p_{s,t,a} \right)= \beta_{0}+{\boldsymbol{\beta}_{\boldsymbol{1}}\boldsymbol{X}}_{\boldsymbol{s,t}}\boldsymbol{+}{\epsilon_{GP}}_{s,t,a}+\epsilon_{ctry_{[s]}}+\epsilon_{s,t,a}+{\epsilon_{GP}}_{a}$$

$$p_{i}= \sum p_{s,t,a}\cdot w_{a}$$

, where $p$ is coverage among 5-x-5-km pixel $s$, year $t$ from 2000 to 2019, and age-group $a$ (i.e., 9-11 months, 1 year, 2 years, 3 years, and 4 years). $\boldsymbol{X}_{\boldsymbol{s,t}}$ are the predicted surfaces from generalized additive models, least absolute shrinkage and selection operator (i.e., LASSO) models, and boosted regression tree models, which are space-time specific only (not age-specific). ${\epsilon_{GP}}_{s,t,a}$ is a correlated space-time-age error term, where the spatial covariance modeled using a Matérn function, temporal covariance modeled as an autoregressive process of order 1 and the age group covariance is also modeled as an autoregressive process of order 1. $\epsilon_{ctry_{[s]}}$ is a country-level random effect, $\epsilon_{s,t,a}$ is a nugget effect to represent observation-specific irreducible error, and ${\epsilon_{GP}}_{a}$ is a correlated age-only error term. After fitting $p_{s,t,a}$, $p_{i}$ was calculated as the sum of age-disaggregated coverage ($p_{s,t,a}$) estimates multiplied by their respective population cohort weights ($w_{a}$). 5-x-5 km level estimates are aggregated to the second administrative units using population-weighted averages across administrative boundaries. Models are fit using Template Model Builder (TMB, version 1.9.1) in R version 5.4.0.

Estimates of second-dose measles containing vaccine (MCV2) coverage were obtained by using hierarchical models at the second-administrative level. These models leverage global, and country priors on parameter values to fit second-administrative unit trends in MCV2 coverage across time. To ensure MCV2 coverage is lower than MCV1, we modeled a ratio of MCV2/MCV1 coverage using the following equations:

Global

$$C_{t,g}\sim Binomial\left( N_{t,g},p_{t,g} \right)$$

$$logit\left( p_{t,g} \right)= \beta_{0,g}+ \boldsymbol{\beta}_{1,g}*Spline\left( t \right)$$

Country

$$C_{t,c}\sim Binomial\left( N_{t,c},p_{t,c} \right)$$

$$logit\left( p_{t,c} \right)= \beta_{0,c}+ \boldsymbol{\beta}_{1,c}*Spline\left( t \right)$$

$$\boldsymbol{\beta}_{\left( x \right),c} \sim N(\boldsymbol{\beta}_{\left( x \right),g}, {(\sigma*\theta)}^{2})$$

First-adminsitrative unit

$$C_{t,a1}\sim Binomial\left( N_{t,a1},p_{t,a1} \right)$$

$$logit\left( p_{t,a1} \right)= \beta_{0,a1}+ \boldsymbol{\beta}_{1,a1}*Spline\left( t \right)$$

$$\boldsymbol{\beta}_{\left( x \right),a1} \sim N(\boldsymbol{\beta}_{\left( x \right),a1}, {(\sigma*\theta)}^{2})$$

, where $p$ is the coverage ratio across time $t$ at various geographic levels (i.e., global ($g$), country-specific ($c$), or first-administrative unit-specific ($a1$). $\boldsymbol{\beta}$ values are normally-distributed priors informed by parameters in either previous global or country-specific hierarchical fits. $\theta$ was an additional parameter used to increase or reduce the influence of the priors on modelled estimates. $\theta$ in the country-specific models was 30 and was 3 in the first-administrative unit level models. Our generalized additive models use thin-plate regression splines on our covariates, modified to penalize null space slightly so that the arguments can be shrunk to zero, with starting k=3. Models were fit using a Bayesian meta-regression in R version 5.4.0. Model predictions were made at the second-administrative unit level following fitted values at the first-administrative unit level and using draw-level geospatially-modeled estimates of MCV1. Models were calibrated (in logit space) to results from the Global Burden of Disease Study^2^; methods have been described elsewhere^1^. In short, to ensure the population-weighted national average of coverage from the subnational models were equivalent to national-level coverage estimates, the following calibration was used:

$$logit\left( V_{GBD,t} \right)=logit\left( V_{subnational, t} \right)+k_{t}$$

, where $V_{GBD,t}$ is national-level coverage for time $t$, $V_{subnational, t}$ is the population-weighted national average of coverage from subnational models at time $t$, and $k_{t}$ is a calibration factor for time $t$ that is chosen such that the un-logit-transformed means are equal after the subnational values are adjusted in logit space.

*Supplementary immunization activities*

Estimates of routine immunization (RI) coverage do not include doses administered via campaigns or supplemental immunization activities (SIAs)**.** In order to also account for doses administered through SIAs, we developed a cohort model that estimates “RI + SIA” coverage for MCV1 and MCV2 by age, space and time from 1980 to 2019. This model leverages RI coverage estimates, the number of doses reported to be administered through the SIA^3^, and the relative risk of being vaccinated during a campaign given previous vaccination status. We compute the relative risk or risk ratio (RR) via a meta-analysis of data from children with vaccine cards with parents interviewed during household-based surveys or post-campaign coverage surveys asking information about campaign participation, with similar results to those previously shown^4^. Then, we compute a metric of “campaign-efficiency”, $p$, which is computed at the most geographically granular resolution possible. Below is the derivation of $p$:

$$RR=\frac{p_{vax}|vaccinated}{p_{unvax}|vaccinated}$$

$$p_{vax}|vaccinated= RR* p_{unvax}|vaccinated$$

$$\# of doses administered=(p_{vax}|vaccinated*\# previously vaccinated)+(p_{vax}|unvaccinated*\# previously unvaccinated)$$

$$\# of doses administered= p_{vax}|vaccinated*\# previously vaccinated+ p$$

$$p=\frac{\# of doses administered}{\left( RR*\# previously vaccinated \right)+\# previously unvaccinated}$$

Then, we compute RI + SIA coverage by week, age, and second-administrative unit such that:

*RI + SIA coverage* = $\frac{\left( \left( \# previously vaccinated \right) + p * \left( \# previously unvaccinated \right) \right)}{total pop}$

The cohorting model is run for each subnational unit through 2019 while also accounting for demographic changes (i.e., aging, births, mortality, and migration) using the same methods, assumptions and data inputs to those described for our transmission model (e.g., population surfaces from WorldPop^5^ calibrated to the Global Burden of Disease study^6^).

# **Section 2. Preliminary model results testing reporting rate formulations**

We tested the following reporting rate structures: single reporting rate and regional reporting rates. For each reporting structure, we tested in a sensitivity analysis four different vaccine effectiveness values. For the final model presented in the main text, we selected the model with the best AIC score, which was the regional and age-specific reporting structure with a vaccine effectiveness of 70%. For all models, see Tables A-D for fitted parameter values and log-likelihood values. Additionally, Fig E contains information on corresponding R_0_ values per $\beta$ value. Additional results using median parameter values can found in Figs G-K.

# ***Section 3. Tables***

**Table A.** GATHER checklist.

| **Item #** | **Checklist item** | **Reported in section** |
| --- | --- | --- |
| **Objectives and funding** | | |
| 1 | Define the indicator(s), populations (including age, sex, and geographic entities), and time period(s) for which estimates were made. | Methods (“Final model fitting algorithm” subsection) |
| 2 | List the funding sources for the work. | Per journal requirements for submission, not yet listed |
| Data inputs | | |
| For all data inputs from multiple sources that are synthesized as part of the study: | | |
| 3 | Describe how the data were identified and how the data were accessed. | Methods (“Case notification” subsection) |
| 4 | Specify the inclusion and exclusion criteria. Identify all ad‐hoc exclusions. | Methods (“Case notifications” subsection) |
| 5 | Provide information on all included data sources and their main characteristics. For each data source used, report reference information or contact name/institution, population represented, data collection method, year(s) of data collection, sex and age range, diagnostic criteria or measurement method, and sample size, as relevant. | Methods and Results (“Case notifications” subsections) |
| 6 | Identify and describe any categories of input data that have potentially important biases (e.g., based on characteristics listed in item 5). | Results (“Case notifications” subsections) |
| For data inputs that contribute to the analysis but were not synthesized as part of the study: | | |
| 7 | Describe and give sources for any other data inputs. | Methods (“Case notifications” and “Dynamic model structure” subsections) |
| For all data inputs: | | |
| 8 | Provide all data inputs in a file format from which data can be efficiently extracted (e.g., a spreadsheet rather than a PDF), including all relevant meta‐data listed in item 5. For any data inputs that cannot be shared because of ethical or legal reasons, such as third‐party ownership, provide a contact name or the name of the institution that retains the right to the data. | Data and code availability; please contact study team for information on how to access full dataset. |
| Data analysis | | |
| 9 | Provide a conceptual overview of the data analysis method. A diagram may be helpful. | Fig 2 |
| 10 | Provide a detailed description of all steps of the analysis, including mathematical formulae. This description should cover, as relevant, data cleaning, data pre‐processing, data adjustments and weighting of data sources, and mathematical or statistical model(s). | Methods (“Dynamic model structure” and “Final model fitting algorithm” subsections) |
| 11 | Describe how candidate models were evaluated and how the final model(s) were selected. | Methods ( “Final model fitting algorithm” subsection) |
| 12 | Provide the results of an evaluation of model performance, if done, as well as the results of any relevant sensitivity analysis. | Methods ( “Final model fitting algorithm” subsection) |
| 13 | Describe methods for calculating uncertainty of the estimates. State which sources of uncertainty were, and were not, accounted for in the uncertainty analysis. | Methods ( “Final model fitting algorithm” subsection) |
| 14 | State how analytic or statistical source code used to generate estimates can be accessed. | Data and code availability statement |
| Results and Discussion | | |
| 15 | Provide published estimates in a file format from which data can be efficiently extracted. | Data and code availability statement |
| 16 | Report a quantitative measure of the uncertainty of the estimates (e.g. uncertainty intervals). | Results (“Subnational susceptibility patterns” subsection), Data and code availability statement |
| 17 | Interpret results in light of existing evidence. If updating a previous set of estimates, describe the reasons for changes in estimates. | Discussion |
| 18 | Discuss limitations of the estimates. Include a discussion of any modelling assumptions or data limitations that affect interpretation of the estimates. | Methods (“Model fitting” subsections), Discussion |

**Table B.** Supplemental immunization activities (SIAs) in Ethiopia from 2000 to 2019.

| Year | Activity | Start date | End date | Age | Extent | Target (N) | Campaign coverage (%) |
| --- | --- | --- | --- | --- | --- | --- | --- |
| 2000 | FollowUp | 6/23/00 | Unknown | 9-59 M | Sub-National | 3800000 | 95 |
| 2000 | FollowUp | 12/29/00 | Unknown | 9-59 M | Sub-national | 3026147 | 78 |
| 2001 | FollowUp | 11/1/01 | 12/30/01 | 9-59 M | Sub-National | 2166232 | 76 |
| 2002 | CatchUp | 11/25/02 | 12/4/02 | 9 M-14 Y | Rollover-Nat | 2316214 | 98.3 |
| 2003 | CatchUp | 4/1/03 | 12/1/03 | 6 M-14 Y | Rollover-Nat | 5605502 | 91 |
| 2004 | CatchUp | 3/20/04 | 6/4/04 | 6 M-14 Y | Rollover-Nat | 8835802 | 84 |
| 2005 | CatchUp | Unknown | Unknown | 6 M-14 Y | Sub-National | 198456 | 69 |
| 2005 | FollowUp | 9/1/05 | Unknown | 9-59 M | Rollover-Nat | 1073066 | 92 |
| 2006 | FollowUp | 3/1/06 | 6/1/06 | 9-59 M | Rollover-Nat | 11688720 | 87 |
| 2007 | FollowUp | 11/1/07 | 11/30/07 | 6-59 M | Sub-National | 1117345 | 96 |
| 2008 | FollowUp | 10/31/08 | 12/7/08 | 6-59 M | Sub-National | 11791819 | 92 |
| 2009 | FollowUp | 1/1/09 | 1/31/09 | 6-59 M | Sub-National | 62504 | 93 |
| 2009 | FollowUp | 1/1/09 | 1/31/09 | 6-59 M | Sub-National | 279102 | 95 |
| 2009 | FollowUp | 1/1/09 | 1/31/09 | 6-59 M | Sub-National | 773910 | 86 |
| 2009 | FollowUp | 6/10/09 | 6/17/09 | 6-59 M | Sub-National | 285644 | 93 |
| 2010 | FollowUp | 2/18/10 | 4/30/10 | 6-59 M | Sub-National | 1057327 | 91 |
| 2010 | FollowUp | 10/22/10 | 10/25/10 | 9-47 M | Rollover-Nat | 7656367 | 107 |
| 2011 | FollowUp | 2/18/11 | 2/21/11 | 9-47 M | Rollover-Nat | 774658 | 98 |
| 2011 | Campaign | 10/1/11 | 11/1/11 | 6 M-14 Y | unknown | 7326463 | 96 |
| 2013 | CatchUp | 5/29/13 | 6/5/13 | 9-59 M | National | 11873928 | 98 |
| 2015 | Outbreak Response | 10/19/15 | 1/31/16 | 6-59 M | Sub-national | 4865893 | 106 |
| 2016 | CatchUp | 4/22/16 | 4/28/16 | 6 M-<15 Y | Sub-national | 25706550 | 97 |
| 2017 | FollowUp | 2/23/17 | 3/10/17 | 9 M-14 Y | Sub-national | 22035787 | 96.3 |
| 2017 | FollowUp | 7/29/17 | 8/4/17 | 6-179 M | Sub-national | 2579178 | 98 |

**Table C.** Overview of main parameters for transmission model.

| **Parameter** | **Description** |
| --- | --- |
| G | Gravity matrix |
| P | Population size |
| V | Distance in minutes (from friction surface) |
| K | Mobility matrix (derived from gravity matrix) |
| $\psi$ | Probability of staying home zone in given epiweek |
| Z,y,d, | Zone |
| w | Epiweek |
| $\beta$ | Transmission probability |
| A | Amplitude |
| D | Displacement |
| FOI | Force of infection |
| M | Maternally immune |
| S | Susceptible |
| I | Infected |
| R | Recovered |
| $\rho$ | Reporting rate |
| $\rho_{Q}$ | Regional reporting rate |
| Vax_eff | Vaccine effectiveness |
| $\vartheta$ | Maternal immunity waning |
| $\gamma$ | Recovery rate |

**Table D1**. Testing various starting values for model with regional reporting including fitting vaccine effectiveness. Starting states yielded inconsistent identified parameters (columns bolded).

|  | Starting value 1 | | Starting value 2 | | Starting value 3 | | Starting value 4 | |
| --- | --- | --- | --- | --- | --- | --- | --- | --- |
|  | Start | End | Start | End | Start | End | Start | End |
| $\beta_{max}$ | 0.7 | **0.51** | 0.6 | **0.57** | 0.9 | **0.59** | 0.51 | **0.50** |
| $\beta_{min}$ | 0.6 | **0.43** | 0.5 | **0.39** | 0.8 | **0.56** | 0.43 | **0.42** |
| ${logit(\rho}_{Addis Abeba})$ | -3 | **-5.80** | -5 | **-4.36** | -4 | **-4.70** | -5.8 | **-4.26** |
| $logit(\rho_{Afar})$ | -3 | **-5.26** | -5 | **-5.28** | -4 | **-5.58** | -5.3 | **-5.32** |
| $logit(\rho_{Amhara})$ | -3 | **-5.02** | -5 | **-4.84** | -4 | **-5.07** | -5 | **-4.81** |
| $logit(\rho_{Benshangul-Gumaz})$ | -3 | **-4.26** | -5 | **-5.63** | -4 | **-5.74** | -4.3 | **-5.47** |
| ${logit(\rho}_{Dire Dawa})$ | -3 | **-3.03** | -5 | **-5.91** | -4 | **-6.05** | -3 | **-5.66** |
| ${logit(\rho}_{Gambela})$ | -3 | **-3.13** | -5 | **-3.15** | -4 | **-3.47** | -3.1 | **-3.19** |
| $logit(\rho_{Harari})$ | -3 | **-3.98** | -5 | **-6.8** | -4 | **-6.01** | -4 | **-5.72** |
| $logit(\rho_{Oromia})$ | -3 | **-4.62** | -5 | **-4.45** | -4 | **-4.68** | -4.6 | **-4.51** |
| $logit(\rho_{Somali})$ | -3 | **-4.99** | -5 | **-5.00** | -4 | **-5.30** | -5 | **-4.98** |
| ${logit(\rho}_{SNNP})$ | -3 | **-5.00** | -5 | **-5.12** | -4 | **-5.39** | -5 | **-5.10** |
| ${logit(\rho}_{Tigray})$ | -3 | **-5.57** | -5 | **-5.67** | -4 | **-6.20** | -5.6 | **-5.73** |
| $logit(vaccine$  $effectiveness)$ | 0.5 | **0.63** | 0.8 | **0.53** | 0.6 | **0.00** | 0.6 | **0.62** |

**Table D2.** Testing various starting values for model with regional reporting without fitting vaccine effectiveness with inconsistent results still observed.

|  | Starting value 1 | | Starting value 2 | | Starting value 3 | | Starting value 4 | |
| --- | --- | --- | --- | --- | --- | --- | --- | --- |
|  | Start | End | Start | End | Start | End | Start | End |
| $\beta_{max}$ | 0.7 | 0.65 | 0.6 | 0.81 | 0.9 | 0.81 | 0.51 | 0.51 |
| $\beta_{min}$ | 0.6 | 0.5 | 0.5 | 0.74 | 0.8 | 0.74 | 0.43 | 0.34 |
| ${logit(\rho}_{Addis Abeba})$ | -3 | -4.42 | -5 | -4.49 | -4 | -4.49 | -5.8 | -3.77 |
| $logit(\rho_{Afar})$ | -3 | -5.43 | -5 | -5.58 | -4 | -5.58 | -5.3 | -5.26 |
| $logit(\rho_{Amhara})$ | -3 | -4.83 | -5 | -4.86 | -4 | -4.86 | -5 | -4.68 |
| $logit(\rho_{Benshangul-Gumaz})$ | -3 | -5.58 | -5 | -5.57 | -4 | -5.57 | -4.3 | -4.75 |
| ${logit(\rho}_{Dire Dawa})$ | -3 | -5.86 | -5 | -5.97 | -4 | -5.97 | -3 | -3.48 |
| ${logit(\rho}_{Gambela})$ | -3 | -3.34 | -5 | -3.47 | -4 | -3.47 | -3.1 | -2.71 |
| $logit(\rho_{Harari})$ | -3 | -5.90 | -5 | -6.01 | -4 | -6.02 | -4 | -5.35 |
| $logit(\rho_{Oromia})$ | -3 | -4.48 | -5 | -4.61 | -4 | -4.61 | -4.6 | -4.74 |
| $logit(\rho_{Somali})$ | -3 | -5.11 | -5 | -5.33 | -4 | -5.33 | -5 | -5.13 |
| ${logit(\rho}_{SNNP})$ | -3 | -5.21 | -5 | -5.31 | -4 | -5.31 | -5 | -4.85 |
| ${logit(\rho}_{Tigray})$ | -3 | -5.95 | -5 | -6.09 | -4 | -6.09 | -5.6 | -5.32 |

**Table E.** Models with single reporting rates, with different vaccine effectiveness values with 95% uncertainty interval from bootstrapped samples.

| Vaccine effectiveness | 47% | 70% | 82% | 88% |
| --- | --- | --- | --- | --- |
| Log-Likelihood | -24260.2  (-24617.4,  -23693.4) | -27538.6  (-28058.0,  -26884.6) | -48654.4  (-53637.5,  -36397.1) | -48555.4  (-53289.0,  -44474.3) |
| $\beta_{max}$ | 0.135  (0.133,  0.138) | 0.224  (0.219,  0.247) | 0.597  (0.277,  0.712) | 0.632  (0.400,  0.830) |
| $\beta_{min}$ | 0.102  (0.102,  0.103) | 0.138  (0.135, 0.146) | 0.066  (0.010, 0.256) | 0.219  (0.073,  0.368) |
| $\rho$ | 0.007  (0.007, 0.008) | 0.054  (0.037, 0.058) | 1  (1, 1) | 1  (1, 1) |

**Table F.** Models with regional reporting rates, with different vaccine effectiveness values with 95% uncertainty interval from bootstrapped samples.

| Vaccine effectiveness | 47% | 70% | 82% | 88% |
| --- | --- | --- | --- | --- |
| Log-likelihood | -23865.3  (-24249.8,  -23373.5) | -26218.7  (-26793.5,  -25739.2) | -48416.0  (-53724.5,  -36172.8) | -49346.6  (-53712.6,  -45012.9) |
| $\beta_{max}$ | 0.1349  (0.1336, 0.1374) | 0.2198  (0.2176, 0.2250) | 0.5966  (0.2773, 0.7123) | 0.7116  (0.3990, 0.8332) |
| $\beta_{min}$ | 0.1021  (0.1019, 0.1024) | 0.1385  (0.1366, 0.1400) | 0.0671  (0.0101, 0.2559) | 0.1559  (0.0673, 0.3725) |
| $\rho_{Addis Abeba}$ | 0.0132  (0.0123, 0.0144) | 0.3881  (0.3363, 0.4493) | 1  (1, 1) | 1  (1, 1) |
| $\rho_{Afar}$ | 0.0045  (0.0041, 0.0049) | 0.0303  (0.0242, 0.0368) | 1  (1, 1) | 1  (1, 1) |
| $\rho_{Amhara}$ | 0.0069  (0.0065, 0.0072) | 0.1284  (0.1170, 0.1377) | 1  (1, 1) | 1  (1, 1) |
| $\rho_{Benshangul-Gumaz}$ | 0.0055  (0.0051, 0.0060) | 0.0484  (0.0411, 0.0538) | 1  (1, 1) | 1  (1, 1) |
| $\rho_{Dire Dawa}$ | 0.0042  (0.0036, 0.0046) | 0.1238  (0.0996, 0.1546) | 1  (1, 1) | 1  (1, 1) |
| $\rho_{Gambela}$ | 0.0293  (0.0253, 0.0335) | 0.4827  (0.4010, 0.5626) | 1  (1, 1) | 1  (1, 1) |
| $\rho_{Harari}$ | 0.0045  (0.0039, 0.0052) | 0.0315  (0.0225, 0.0431) | 1  (1, 1) | 1  (1, 1) |
| $\rho_{Oromia}$ | 0.0087  (0.0083, 0.0092) | 0.0432  (0.0404, 0.0458) | 1  (1, 1) | 1  (1, 1) |
| $\rho_{Somali}$ | 0.0065  (0.0060, 0.0070) | 0.0381  (0.0341, 0.0415) | 1  (1, 1) | 1  (1, 1) |
| $\rho_{SNNP}$ | 0.0063  (0.0060, 0.0066) | 0.0639  (0.0588, 0.0694) | 1  (1, 1) | 1  (1, 1) |
| $\rho_{Tigray}$ | 0.0026  (0.0024, 0.0027) | 0.0818  (0.0742, 0.0911) | 1  (1, 1) | 1  (1, 1) |

# ***Section 4. Figures***

**Fig A.** Model fit (blue) compared to aggregated quarterly measles cases (pink) in Bale, Ethiopia among 0-to-4-month-olds by quarters.

Models suggested poor fit as misaligned aggregated peaks yielded large penalties to likelihood during evaluations. Bale zone was chosen as an illustrative example. Quarter 1 is the beginning of 2013, quarter 10 being mid-2015, quarter 20 is the beginning of 2018 and quarter 30 is the end of 2019.


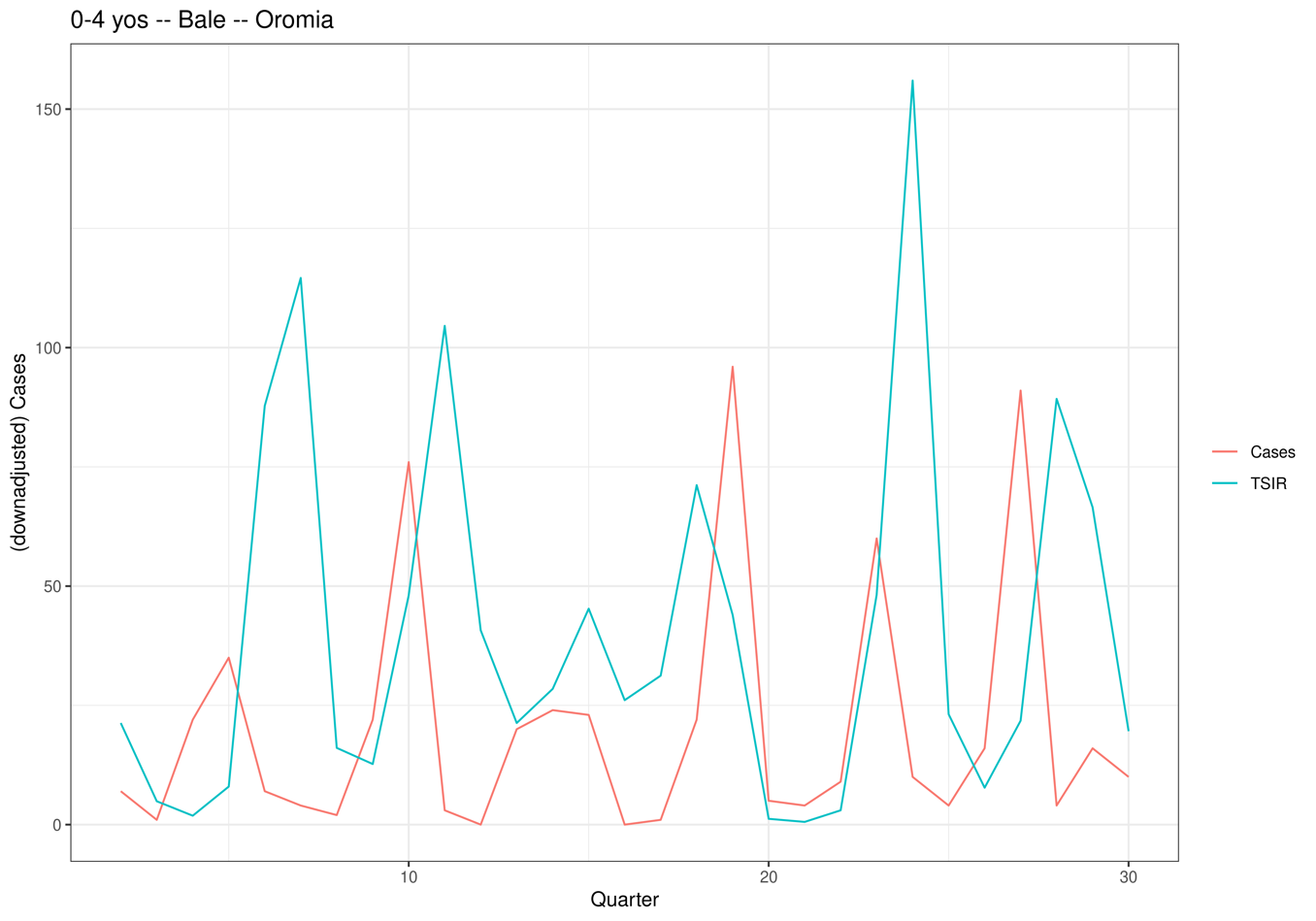


**Fig B.** National-level proportion of the population in each compartment by age group in model with assumed 93% vaccine efficacy.

Models with assumed 93% vaccine efficacy, without any other assumptions or information on vaccine effectiveness, yielded little to no transmission in later years as seen in the bottom left panel of the Infected compartment. Week 0 is the beginning of 1980, week 500 is mid-1989, week 1000 is mid-1998, week 1500 is mid 2008, and week 2000 is mid-2017. The end of the time series is the end of year 2019.


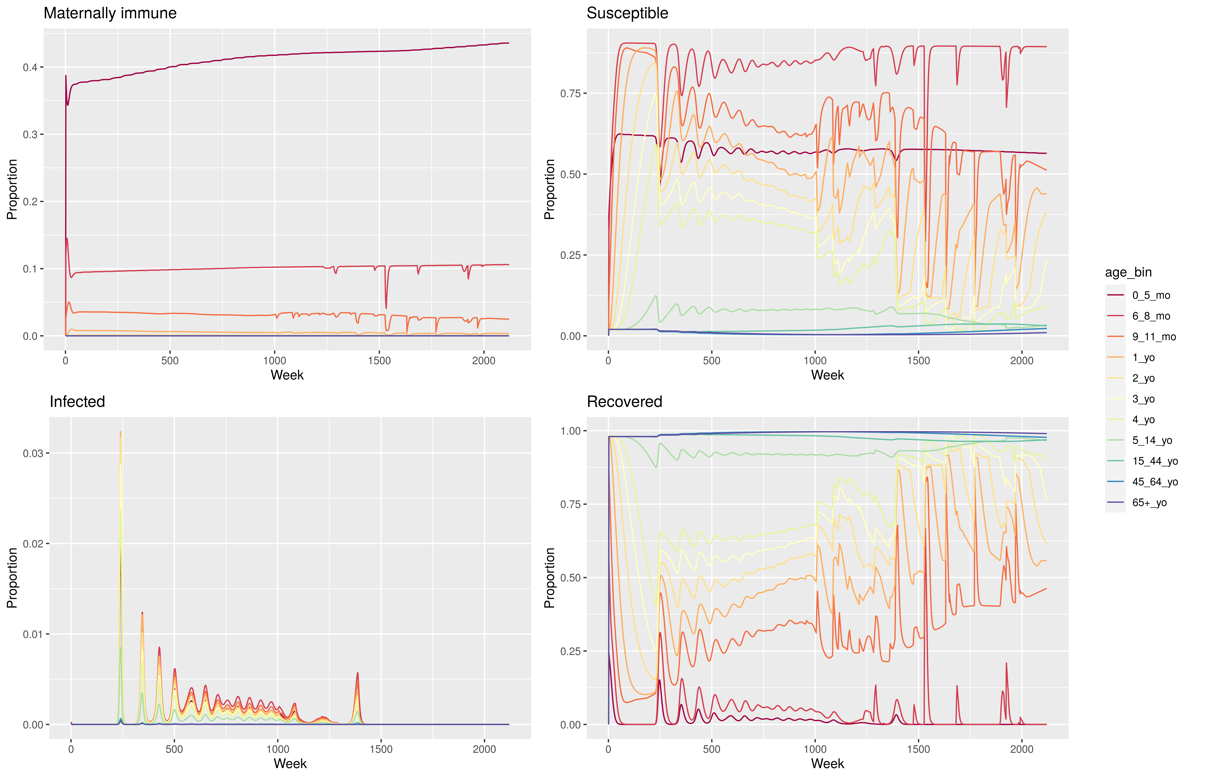


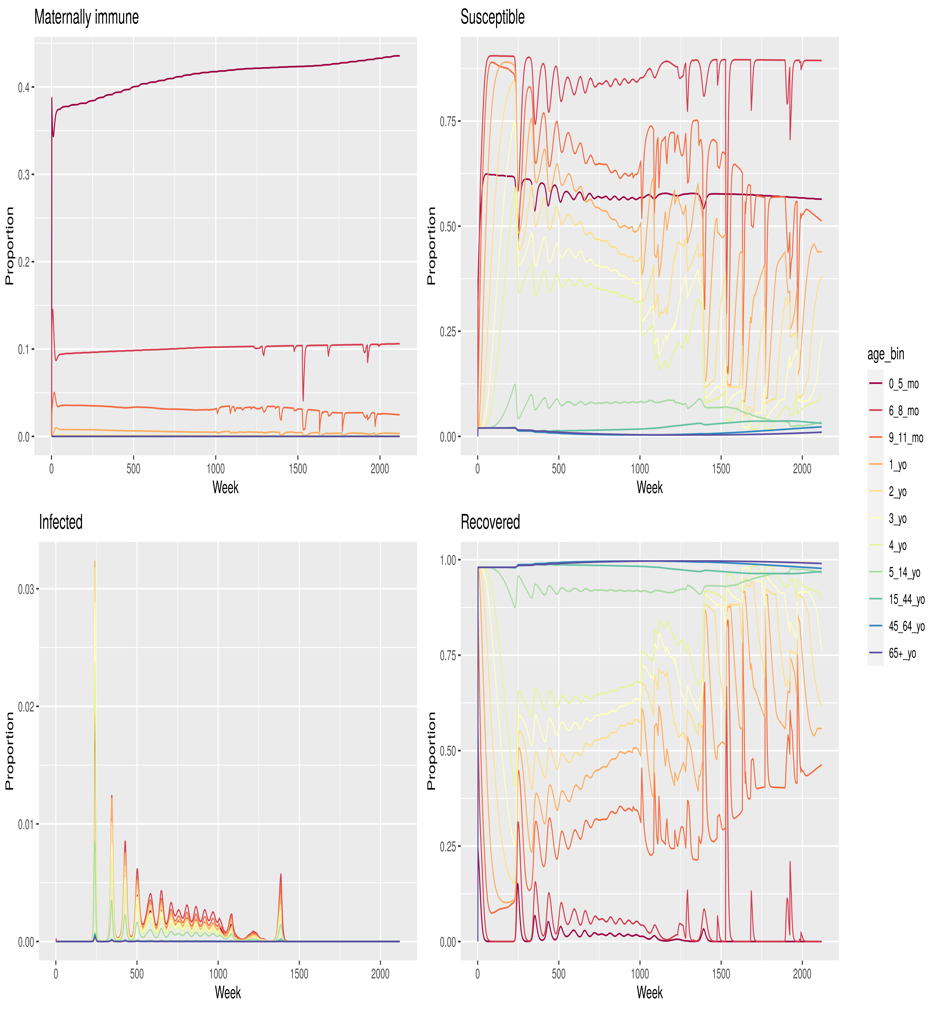


**Age**

0-to-5-months-old

6-to-8-months-old

9-to-11-months-old

1-year-old

2-years-old

3-years-old

4-years-old

5-to-14-years-old

15-to-44-years-old

45-to-64-years-old

65+-years-old

**Fig C.** Trace plots from MCMC model using parallel tempering.

Trace plots of over 12000 samples accepted less than 1% of proposed samples, in lowest temperature chain of parallel tempering. For each parameter, different y-axes were used. The following panels are representative of the following parmaters:

- Maximum transmission probability: max_beta
- Minimum transmission probability: min_beta
- Logit of reporting rate for Addis Abeba: logit_rho1
- Logit of reporting rate for Afar: logit_rho2
- Logit of reporting rate for Amhara: logit_rho3
- Logit of reporting rate for Benshangul-Gumaz: logit_rho4
- Logit of reporting rate for Dire Dawa: logit_rho5
- Logit of reporting rate for Gambela: logit_rho6
- Logit of reporting rate for Harari: logit_rho7
- Logit of reporting rate for Oromia: logit_rho8
- Logit of reporting rate for Somali: logit_rho9
- Logit of reporting rate for SNNP: logit_rho10
- Logit of reporting rate for Tigray: logit_rho11
- Logit of vaccine effectiveness: logit_vax_efficacy


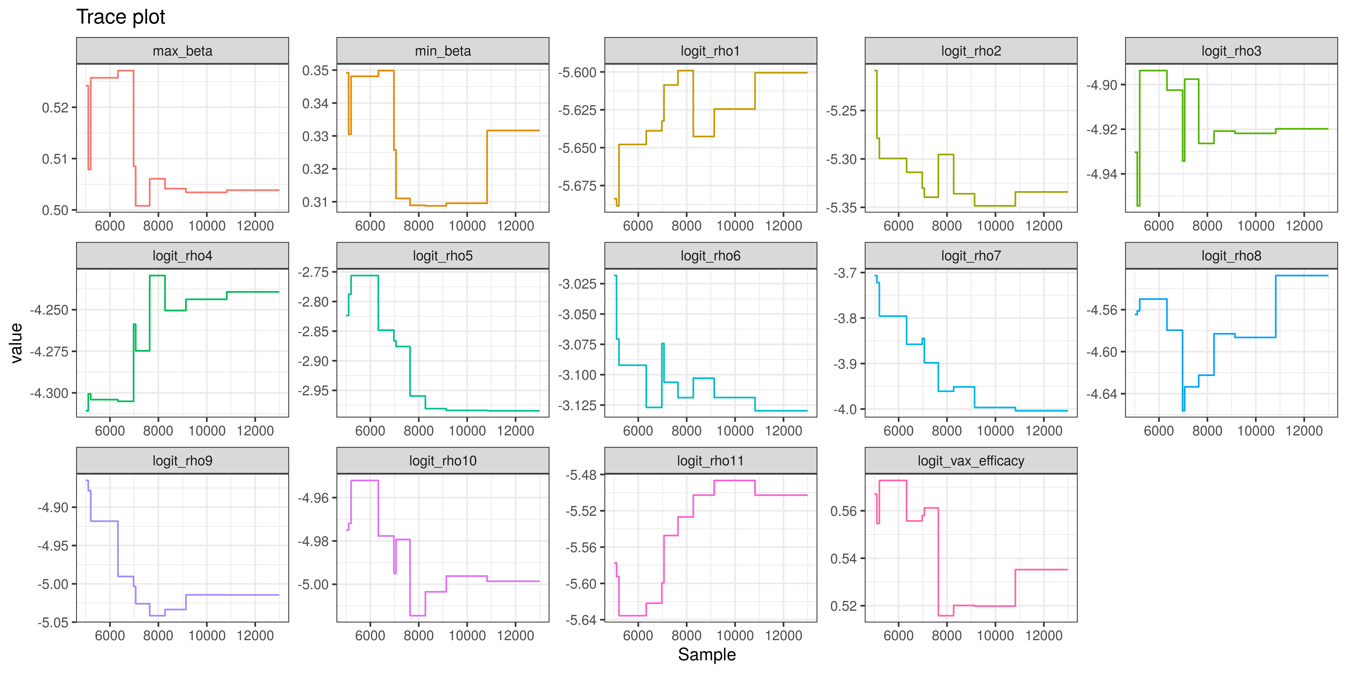


**Fig D.** Reported cases across zone-weeks from 2013 to 2019.

**
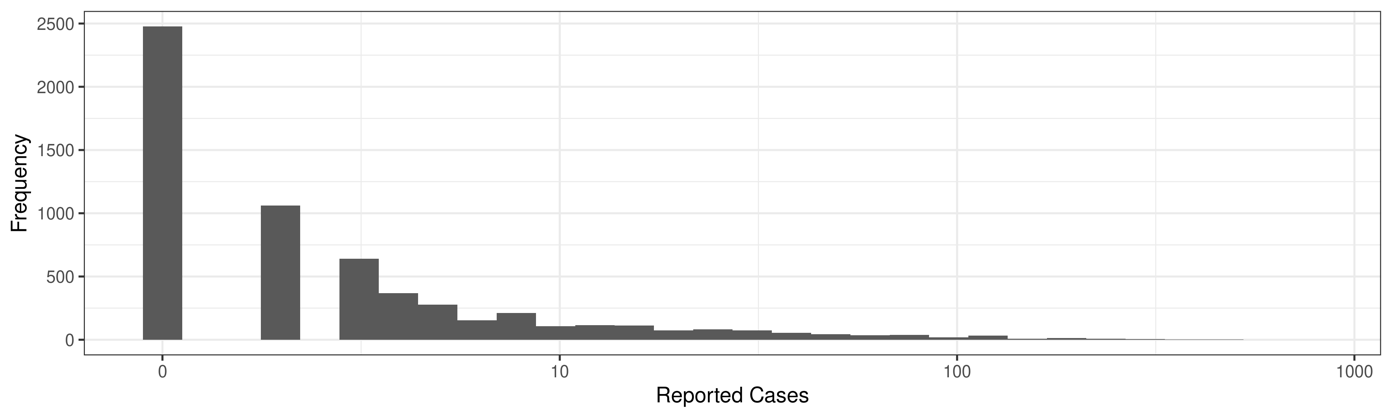
**

**Fig E.** R_o_ value by transmission probability parameters ($\beta$).


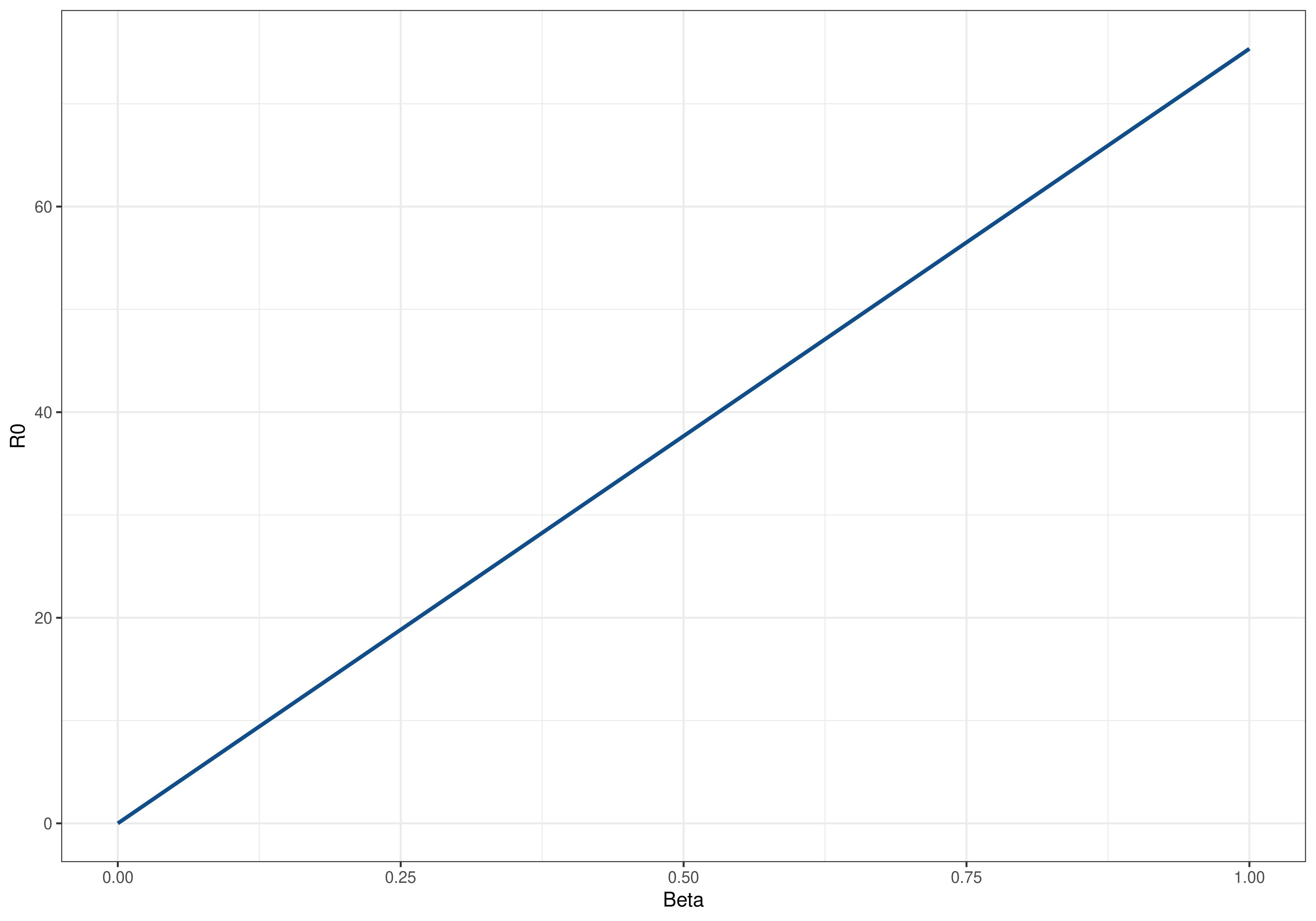


**Fig F.** Block coordinate descent iterations for each parameter from model selected with best fit. Each line represents one bootstrapped sample (n = 100 per parameter).


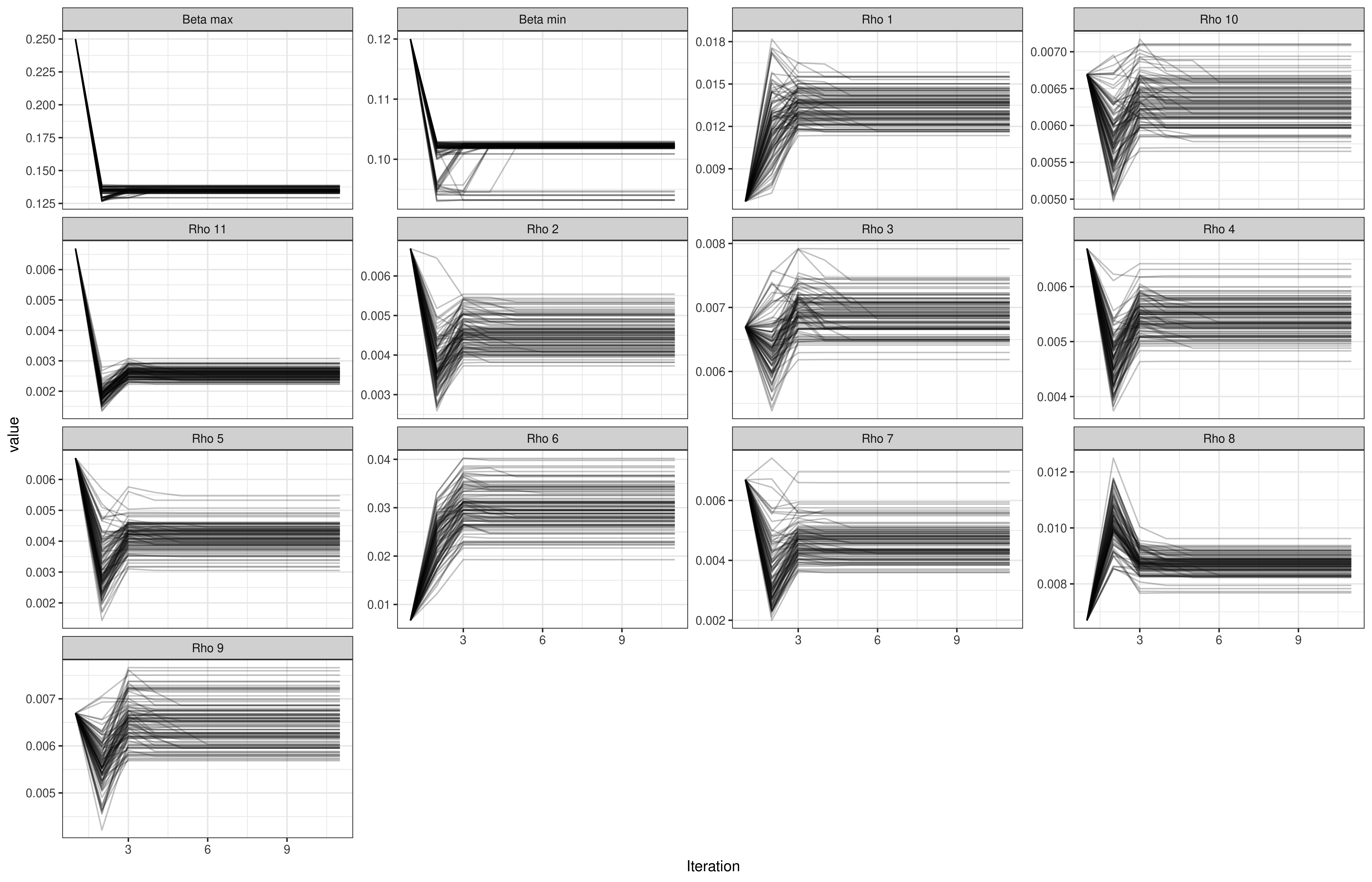


**Fig G.** Smoothed reported suspected measles incidence among 5-to-9-year-olds (green) compared to estimated incidence adjusted for reporting (black) from best model fit across each zone in weeks from 2013 to 2019.


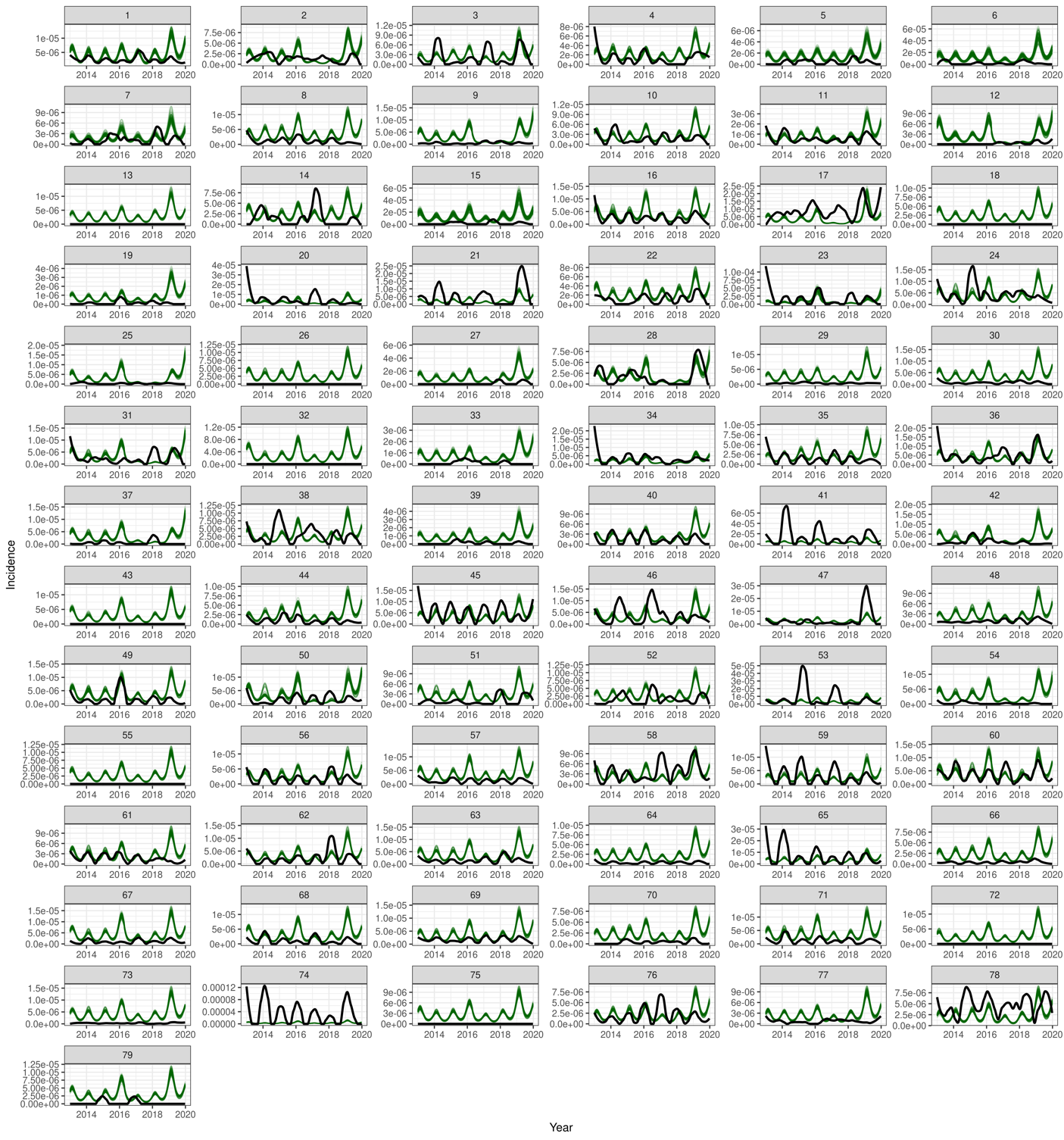


**Fig H.** Smoothed reported suspected measles incidence among 10-to-14-year-olds (orange) compared to estimated incidence adjusted for reporting (black) from best model fit across each zone in weeks from 2013 to 2019.


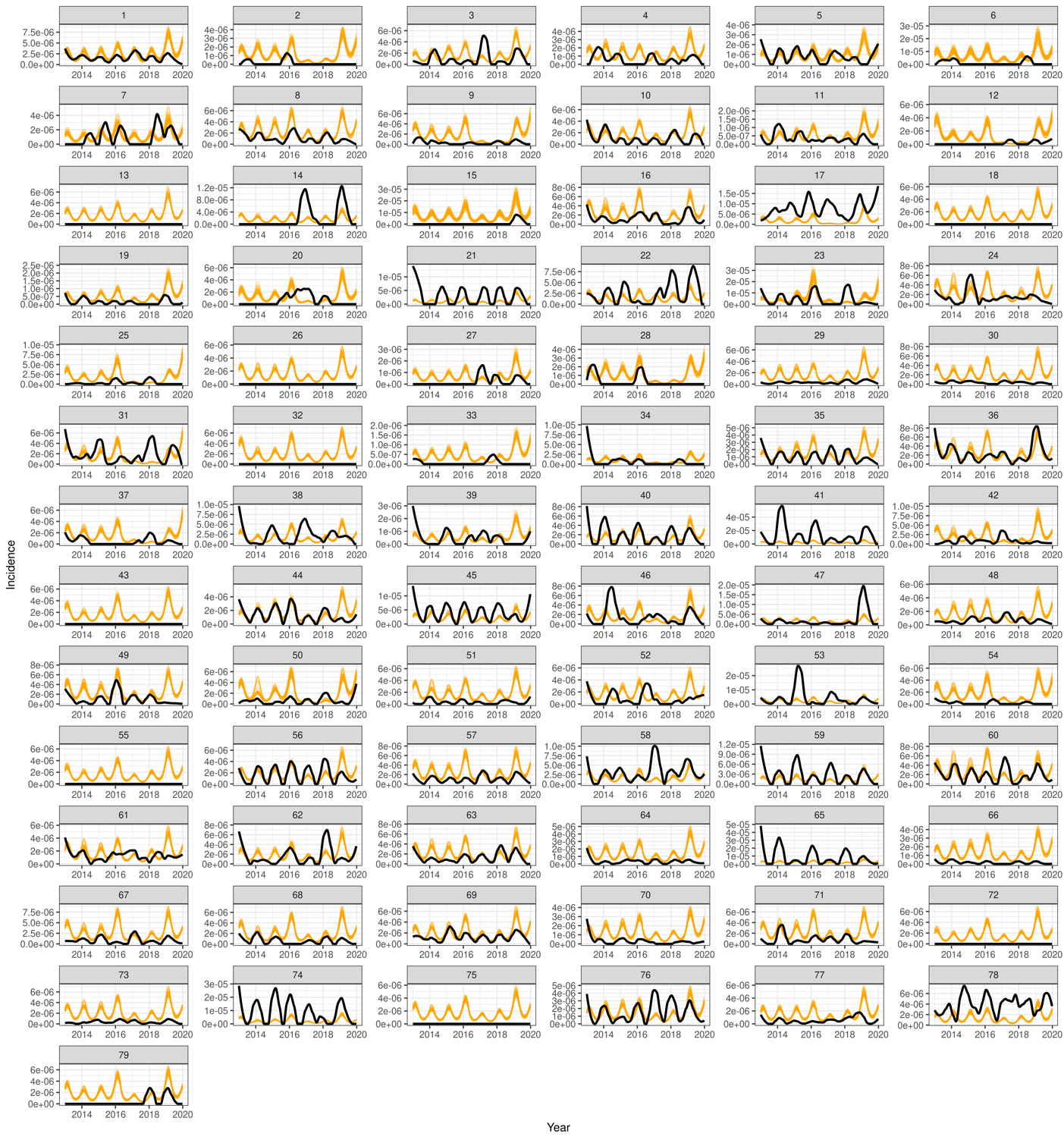


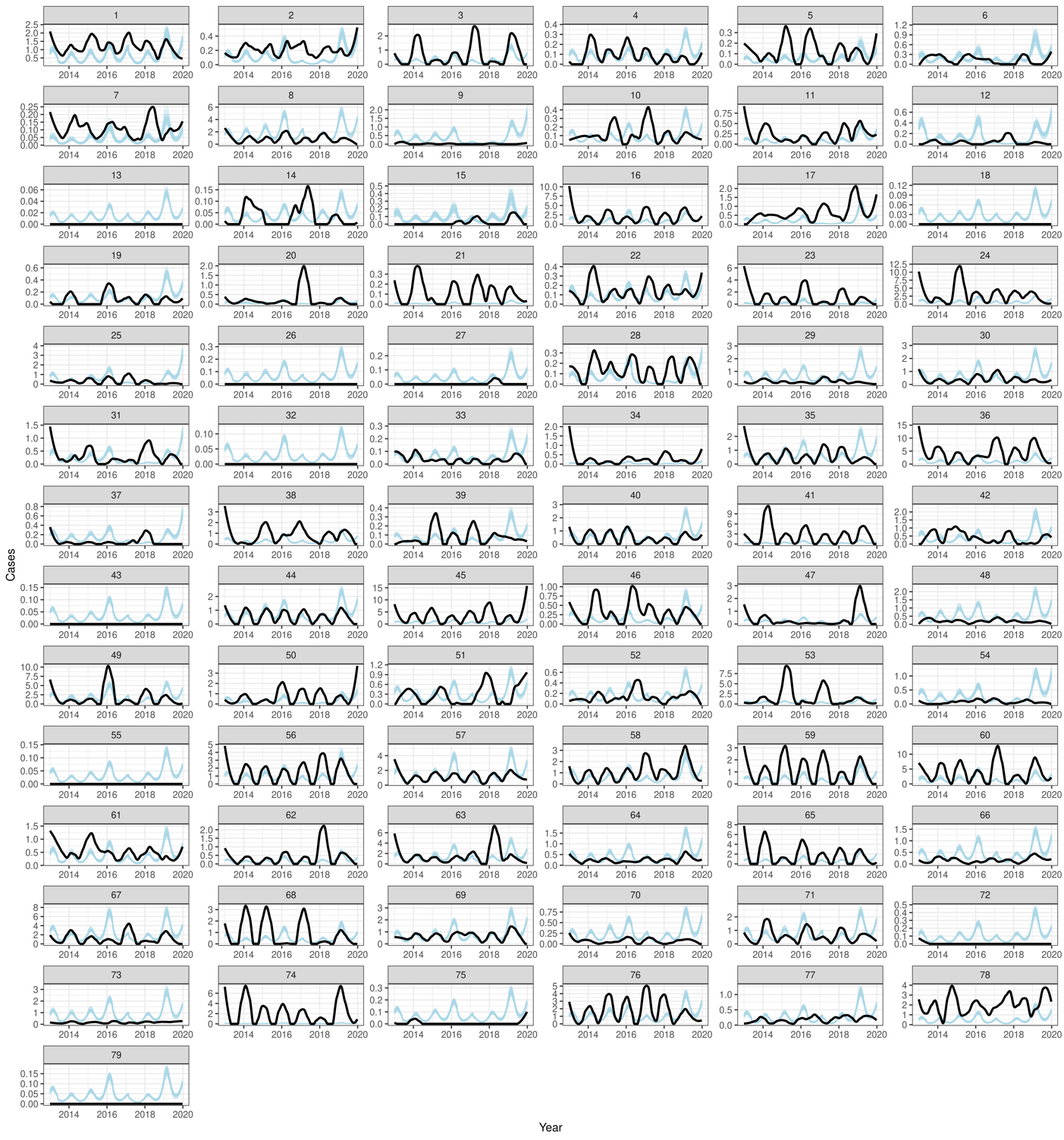
**Fig I.** Smoothed reported suspected measles cases among 0-to-4-year-olds (light blue) compared to estimated incidence adjusted for reporting (black) from best model fit across each zone in weeks from 2013 to 2019.

**Fig J.** Smoothed reported suspected measles cases among 5-to-9-year-olds (green) compared to estimated incidence adjusted for reporting (black) from best model fit across each zone in weeks from 2013 to 2019.

*
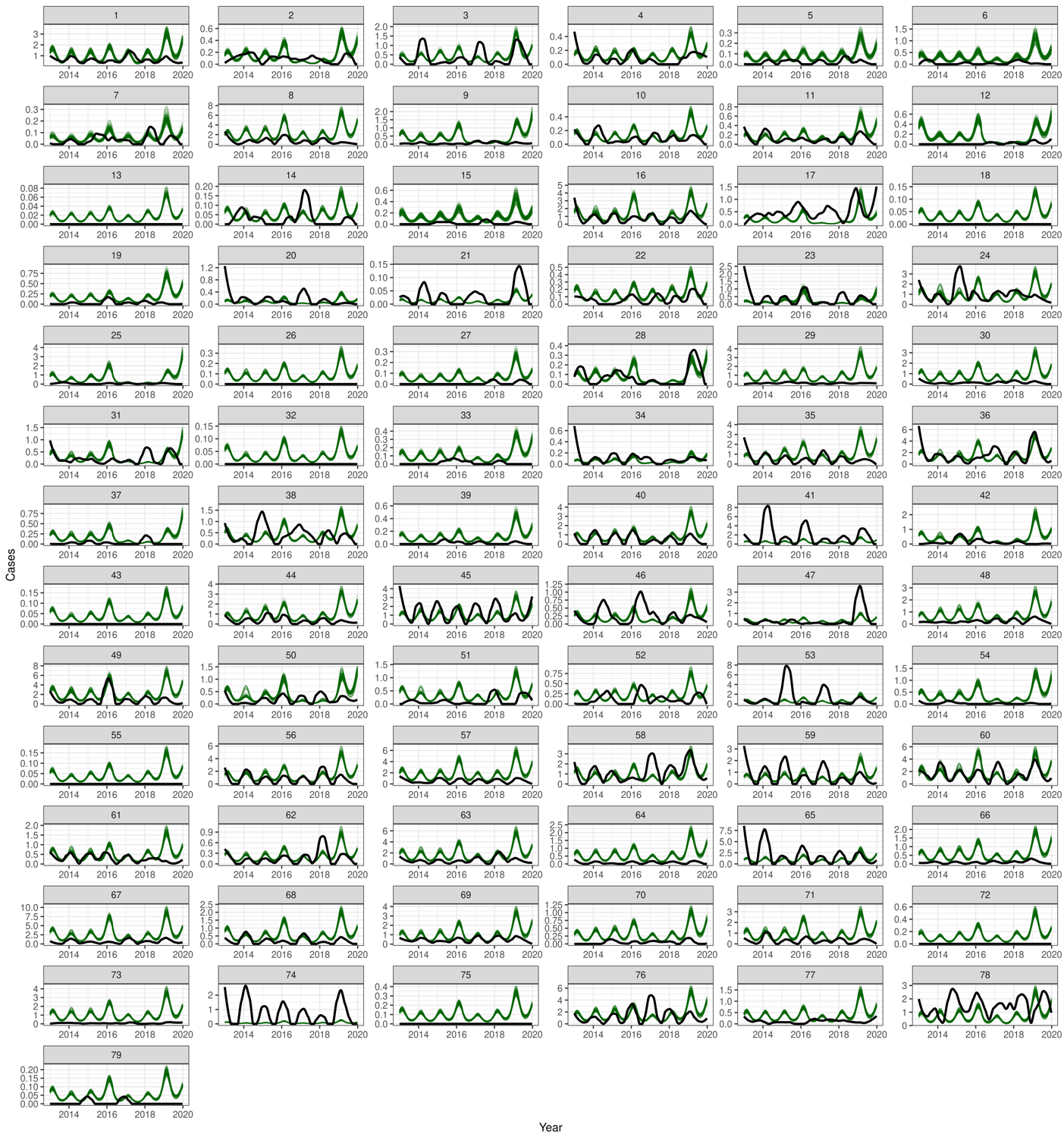
*

**Fig K.** Smoothed reported suspected measles cases among 10-to-14-year-olds (orange) compared to estimated incidence adjusted for reporting (black) from best model fit across each zone in weeks from 2013 to 2019.

*
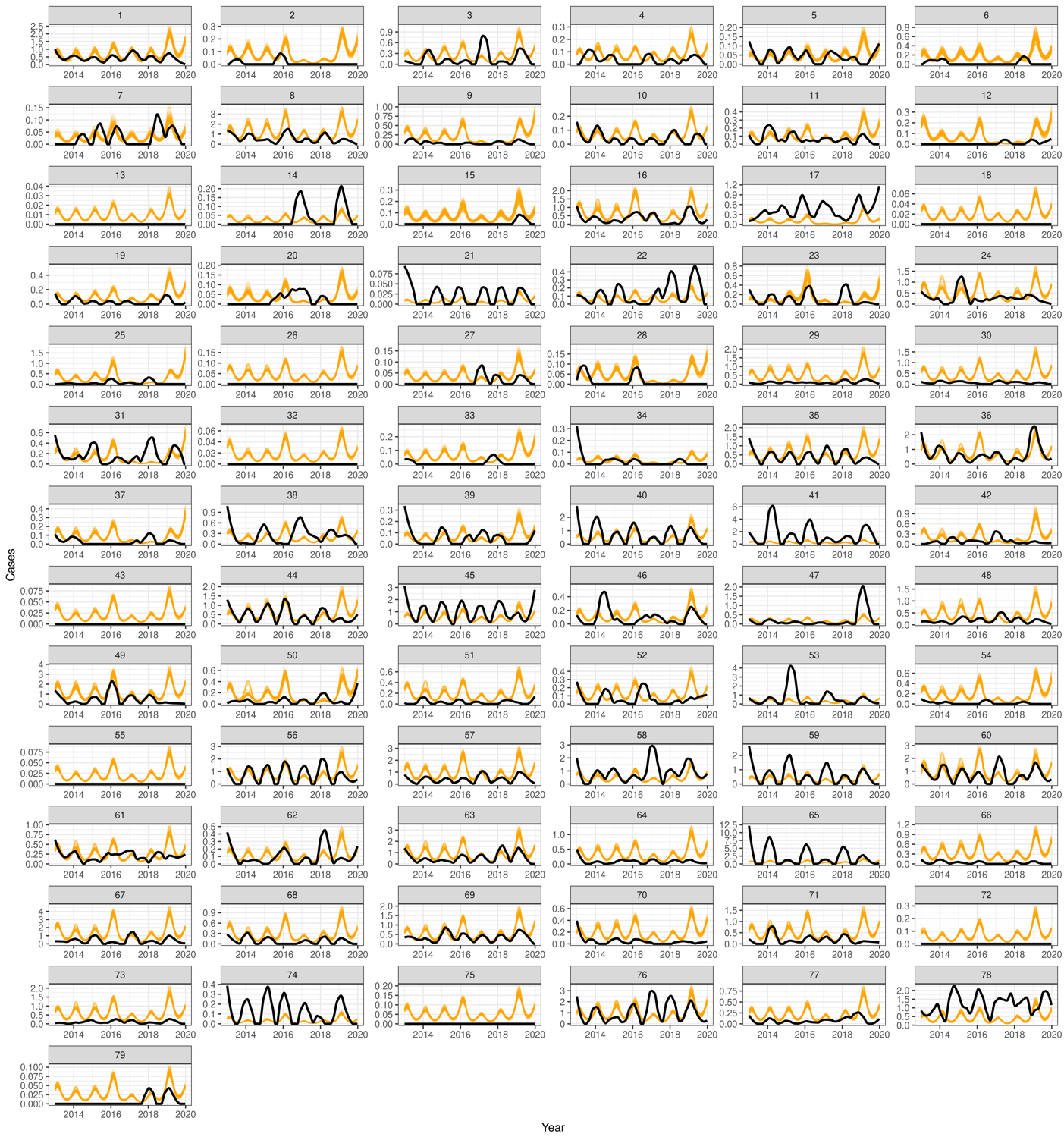
*

# ***Section 6. References***

1. Mapping routine measles vaccination in low- and middle-income countries. *Nature* 2021; **589**(7842): 415-9.

2. Measuring routine childhood vaccination coverage in 204 countries and territories, 1980-2019: a systematic analysis for the Global Burden of Disease Study 2020, Release 1. *Lancet* 2021; **398**(10299): 503-21.

3. World Health Organization. Immunization data. 2023. <https://immunizationdata.who.int/listing.html>.

4. Portnoy A, Jit M, Helleringer S, Verguet S. Impact of measles supplementary immunization activities on reaching children missed by routine programs. *Vaccine* 2018; **36**(1): 170-8.

5. Tatem AJ. WorldPop, open data for spatial demography. *Sci Data* 2017; **4**: 170004.

6. Global burden of 369 diseases and injuries in 204 countries and territories, 1990-2019: a systematic analysis for the Global Burden of Disease Study 2019. *Lancet* 2020; **396**(10258): 1204-22.
