# Supplementary material for: Fitting dynamic measles models to subnational case notification data from Ethiopia: Methodological challenges and key considerations
Source: PLoS Comput Biol. 2025 Apr 16;21(4):e1012922. doi: 10.1371/journal.pcbi.1012922 (PMC12002527; doi:10.1371/journal.pcbi.1012922)
Supplement: Attachment — Submitted filename: renamed_24ab4.pdf [file pcbi.1012922.s002.pdf]

Review of **“Exploring the utility of subnational case notifications in fitting dynamic measles models in Ethiopia”** 2024-04-12

Comments to authors

This article considers a valuable, yet challenging, issue, of identifying subnational areas of measles susceptibility in high-burden settings. Results from these models could be highly beneficial to plan impactful vaccination campaigns and reduce preventable measles burden. Overall, I found the manuscript to be very promising: as the authors note, there are a number of challenges to navigate to design and fit such models. I felt that the authors made useful contributions presenting and discussing these challenges and how to overcome them.

However, on first-reading of the manuscript, I found the positioning of the paper inconsistent between sections: it unclear whether it was aiming to be a methods paper (focussing on the methodological contributions and challenges of the approach in general) or a paper about the trends in subnational measles susceptibility in Ethiopia. My main suggestion for improvement would be to focus the paper on one of these only: given the significant effort made on overcoming the methodological challenges, I would suggest focussing on this part, using measles in Ethiopia as a case study / motivating example, thereby bringing the best parts of the research to the forefront; I have tried to make specific comments to that effect below.

Main comments

1. **Abstract / authors’ summary / introduction.** I felt each of these seemed to be telling a different story, rather than all setting the scene for the same paper. Can the authors make changes so that these three sections are more cohesive, e.g. mentioning the data and model fitting challenges (currently only in the authors’ summary).
2. **Introduction.**
    a. I felt that this focussed too much and too early on Ethiopia, when in fact little of the paper was focussed on Ethiopia specifically. I would suggest that the authors restructure the introduction to introduce first the problem (subnational measles susceptibility estimation) and application (targeted vaccination), then the challenges (case reporting patterns and model fitting difficulties, etc.) and finally present Ethiopia as a case example.
    b. To highlight that this question/model is relevant more broadly, can you also add 1-2 sentences on other settings where this would be relevant.
3. **Methods.**
    a. Again, I would suggest that the authors restructure to present the general model first, then the data and model fitting considerations (below).
    b. Some of the “Model fitting considerations” are more considerations about the data. I think it would be clearer if the authors split the “Model fitting considerations” into two: one on the data (reporting patterns etc.) and one on the model fitting proper (collinearity, steep likelihood surface).
4. **How can results from this model be validated/evaluated?** There are some ideas when discussing the results from the Ethiopia data (comparing model-estimated susceptibility to percentage unvaccinated (1 - MCV1)), but I think it might be useful to draw these out more explicitly. Additionally, are there any other metrics the authors

could use to validate part or all of the results, and what (if any) are the limitations or biases of these? For example, can the authors compare their estimates to the serosurvey data?

5. **Discussion.** More discussion on the limitations to practical use in this, or other, contexts e.g. data requirements, computing requirements? Are there other practical issues (e.g. other reporting artefacts) that might need to be addressed that didn't come up for Ethiopia?

Minor comments

6. Subnational scale. Is admin-2 small enough to identify subnational areas of susceptibility, and is this the same scale on which vaccination campaigns are organised?
7. Sporadic case reporting. The authors note "it was difficult to tell from the data whether the zones that reported alternating weeks with zero cases represent the total sum of cases across two weeks or whether these are missing other values that were not reported". Can any of the co-authors in Ethiopia confirm this, and perhaps provide any explanation as to why this happens?
8. Vaccine coverage estimates: can you clarify in the main text whether these estimates are based on administrative coverage, or coverage surveys?
9. Model notation: α is used for both the amplitude in the definition of β_i, and in the definition of the force of infection (FOI).
10. Parameter values. Why have you chosen the overdispersion parameter $\varphi = 5$ in the definition of reported cases?
