# Supplementary material for: Fitting dynamic measles models to subnational case notification data from Ethiopia: Methodological challenges and key considerations
Source: PLoS Comput Biol. 2025 Apr 16;21(4):e1012922. doi: 10.1371/journal.pcbi.1012922 (PMC12002527; doi:10.1371/journal.pcbi.1012922)
Supplement: Attachment — Submitted filename: eth_case_fitting_rtr_20240626_CLEAN.docx [file pcbi.1012922.s004.docx]

Reviewer #1:

Thank you for the opportunity to review this manuscript. The authors wish to understand the risk of potential index cases at subnational level to determine where to deploy vaccination. The specific vaccination strategy to be considered in response to these cases is not mentioned but given the data sources used is likely to be targeted follow up mass vaccination rather than pulse vaccination. The authors focus on computational considerations that are to be made in fitting and estimating from their model. The main outcome of interest is the number of susceptible persons at various administrative levels.

Measles is an infectious disease of public health concern as a) it requires a very high vaccination coverage to achieve herd immunity (normally in the 95 per cent range), b) it has a high basic reproduction number (usual estimates are around 13), and c) it can cause immune amnesia putting survivors more at risk of other infections afterwards. However, measles (exposure >and< survival or vaccination) should confer lifelong immunity (which allows for simplifying assumptions in a modelling approach, e.g. there need be no flows from removed back to susceptible) and it is only assumed to have a human reservoir making it a candidate for elimination/eradication. There is a dependency in vaccination that needs be captured, namely that second dose is contingent on having received first dose.

The WHO immunisation data portal suggests the routine measles immunisation schedule for an Ethiopian infant is first dose at nine months and second dose at fifteen months. I unfortunately do not know much else about the situation in Ethiopia, but I believe it is bordered by countries with a certain amount of instability whereby regions near the borders could be of particular concern.

With some improvements, I believe the manuscript can become fit for publication. I would be willing to review a revised version of the manuscript. I do not consent to this review being unblinded.

We thank the reviewer for their detailed feedback and for the opportunity to improve our manuscript.

**Wording I found particularly pleasing to read:**
The first two sentences of the abstract set the scene and provide the motivation for the work in a nice manner. The first sentence of the paragraph on line 136 is nice as well as the last sentence in the paragraph on line 513.

We thank the reviewer for their support.

I would suggest updating the notation to ensure avoiding the same symbols for different things, examples:
* g_zy = P_z P_y / f_{zy} (avoids mixup of i)
* m_zy = \begin{cases} (avoids mixup with M compartment)
\frac{g_{zy}(1-\xi)}{\sum_y g_{zy}} & z\neq y \\ (avoids mixup with theta used in supp. inf. and D used for phase)
\xi & z = y \\
\end{cases}
* \xi \in [0, 1], \xi \equiv 0.99
* \beta_w = A \sin\left(\frac{2\pi w}{52} + 2\right) + D, w = \text{week} (avoids mixup of i and a)
* FOI_{adw} = \beta_w \sum_c \sum_y (W_{acwy} m_{zy} I^\alpha_{w-1,cz}, \alpha \in [0, 1], \alpha \equiv 0.99

We thank the reviewer for their helpful suggestions. We have updated our notation for all equations to be internally consistent and distinct across variables. The following notation across the manuscript has been updated as follows:

- $z$ and $y$ to describe zones, $V$ for distance, $K$ for mobility matrices, and $\psi$ for the probability of people staying in their home district
- $w$ for week
- $A$ for amplitude
- $Q$ for region and regional reporting rates

It may be beneficial to the authors as well as the reader to provide an overview table with the variables used and their meaning as can be found in engineering disciplines. Reading the manuscript backwards may help the authors realise where terminology varies (e.g. time step and week) and allow them to harmonise it.

We have added the following table to our appendix (now Table 3) to guide readers on the various parameter values used in the main transmission model, and reference in the main text to this table on lines 273-274 that read as:

“An overview of our modelling structure can be found in Figure 1 and an overview of main parameters in Appendix Table 3.”

| **Parameter** | **Description** |
| --- | --- |
| G | Gravity matrix |
| P | Population size |
| V | Distance in minutes (from friction surface) |
| K | Mobility matrix (derived from gravity matrix) |
| $\psi$ | Probability of staying home zone in given epiweek |
| Z,y,d, | Zone |
| w | Epiweek |
| $\beta$ | Transmission probability |
| A | Amplitude |
| D | Displacement |
| FOI | Force of infection |
| M | Maternally immune |
| S | Susceptible |
| I | Infected |
| R | Recovered |
| $\rho$ | Reporting rate |
| $\rho_{Q}$ | Regional reporting rate |
| Vax_eff | Vaccine effectiveness |
| $\vartheta$ | Maternal immunity waning |
| $\gamma$ | Recovery rate |

**Additional comments:**
Line 108: Providing the numbers for the other years in the study period (i.e. national MCV1 coverage for 2013 to 2018, both years included) will assist the reader in understanding the situation and will support and quantify the statement on line 111: “increased national MCV1 coverage”

This sentence has been revised to now read as follows:

“In 2019, Ethiopia reached 59.0% coverage for the first-dose of any measles-containing vaccine (MCV1) nationally, albeit with substantial subnational coverage heterogeneity, compared to 58.8% coverage in 2013.”

Line 116: Providing a population estimate will help the viewer understand the magnitude of this number

To give additional context and include estimates for total population and population of under-fives, we have revised the following sentence (lines 223-226) to read as:

“In 2017, Ethiopia executed two subnationally-targeted measles campaigns, or supplemental immunization activities (SIAs), in March and August which combined delivered over 23,750,000 vaccine doses (with the 2017 total population size of approximately 100,000,000, and approximately 15,000,000 children under age five).”

Line 123: It is unclear why the United States is being appealed to here and I suggest this either be explained or removed. “Different strategies” is also vague and could be improved by considering specific strategies, e.g. pulse vaccination is often used for measles, but ring vaccination could also be used

The following sentence (lines 123-128) has been revised to remove specific reference to the United States and also discuss alternative strategies beyond campaigns and now reads as:

“Alternatively, high coverage settings with pockets of persistent transmission among communities without explicit geographic division (e.g., among specific social networks), might need different strategies, such as strengthening of health systems and health service provisions as well as tailored communication efforts and discussions of the risks and benefits of vaccination, to increase coverage beyond the use of campaigns.”

Line 133: If serosurveys are not being used in the work, I would remove this sentence

As the other reviewer has requested a discussion of possible mechanisms for validation, we have kept this sentence as is.

Line 153: This information could perhaps be placed in a table with additional information such as study period and location considered

Mentioning these articles had the intention of providing a brief insight into the body of work that has already been explored – and was not meant to describe a comprehensive review. Therefore, to not mislead readers to perceiving this comprehensiveness, we have elected to not include an additional table.

Line 167: I would say “compartmental modelling” rather than “traditional modelling”

This has been revised (lines 202-203) and now reads as:

“To address these methodologic gaps, research exploring new applications to compartmental modelling approaches that incorporate information from case notifications is needed.”

Line 180: Perhaps a table will help the reader understand the administrative divisions of Ethiopia, something like:
+--------------------------------------+--------+
| Level | Number |
+--------------------------------------+--------+
| Country | 1 |
+--------------------------------------+--------+
| Regional states and chartered cities | ~11 |
+--------------------------------------+--------+
| Zones | ~79 |
+--------------------------------------+--------+
| Districts/woredas | ~700+ |
+--------------------------------------+--------+

Instead of an additional table, we have included the following sentence (lines 236-238) within the first paragraph of the methods section to guide readers on the administrative divisions of Ethiopia:

“In Ethiopia, there are 11 regions (i.e., first administrative units), 79 zones (i.e., second-administrative units), and over 700 districts (i.e., third administrative units).”

Line 181: Abbreviation never used again after introduction so not needed

We have revised to remove the abbreviation (GADM) within the sentence (lines 238-240) to now reads as:

“For each subnational location reported within the case data, we identified the most appropriate corresponding zone (i.e., second-administrative division level, n = 79) based on the Database of Global Administrative Areas administrative boundaries.”

Line 189: What does the spatial autocorrelation of cases look like at zone level?

Among cases per 100k persons reported across 0-14 year olds throughout 2019, there is little spatial autocorrelation (Moran’s I = -0.019, p=0.5584) at the zone-level, as evident in the map to the right.

Line 192: Abbreviation SIA is already introduced in the introduction section

We thank the reviewer for noting this duplication and have revised this sentence (lines 251-252) to now read as follows:

“We used MCV1 and MCV2 vaccine coverage estimates from either routine immunization (RI) or SIAs.”

Line 195: Perhaps the authors should note that “epiweek” is an ISO week shifted one day backwards such that the week starts on Sunday rather than Monday as this might not be common knowledge depending on the cultural background of the reader

We added a description of epiweeks slightly earlier in the manuscript (in the section that introduces the case notifications – as their reporting by epiweek is the rationale for using them overall). Lines 233-238 have been revised to now read as:

“We obtained subnational case notifications of suspected measles from across Ethiopia from 2013 (the first year that they were available) to 2019 in 5-year age bins (e.g., 0-4-year-olds, 5-9-year-olds, etc.) that were collected through routine surveillance systems by epiweek (i.e., ISO week shifted one day to begin on Sunday). In Ethiopia, there are 11 regions (i.e., first administrative units), 79 zones (i.e., second-administrative units), and over 700 districts (i.e., third administrative units).”

Line 197: The “additional details” also references previous work so please at least provide a short description. Not all readers may be able to access the Nature reference

We appreciate the need to clearly communicate the methods used to generate coverage estimates, while also note that fully describing the complex models used to generate these estimates would not be possible in this manuscript. We have previously published multiple papers(1,2) on this underlying method for readers that are interested in better understanding the coverage-related methods (currently cited as manuscript references 1 and 2) . We note also that each of these manuscripts is fully open-access, so all readers should be able to fully access these references and the accompanying supplemental material. However, to provide additional context for all readers, as the reviewer suggested, we have added the following brief explanation to the methods section (lines 257-266) that now reads as:

“In short, household-based survey data on MCV1 and MCV2 coverage are geolocated to the most geographically granular level possible. For RI MCV1 coverage, we used a model-based geostatistical framework to estimate coverage under the assumption that coverage is more similar in years and locations that have similar covariate patterns and in years, locations and age groups that are closer across time, space and age. For RI MCV2 coverage, less data was available to include in models, as MCV2 was only introduced in Ethiopia in 2019. Therefore, we used a hierarchical model at the zone level. For combined RI and SIA coverage, we developed a model tracking cohorts through time, accounting for RI coverage increases along with doses reported to be administered during SIAs.”

Line 200: Why is the model relegated to supp. inf. rather than included in the main manuscript? Please add the model equations also as it is unclear why no model equations are given and flows in the diagram are not given parameters

The figures related to the main model have all been moved to the main text. Now main text Figure 2, i.e., the transmission model flow diagram, has also been updated with appropriate parameters. All modeling equations have been already given in the main text on lines 285-320 and lines 359-390. We have additionally specified waning and recovery rates in Figure 2 and Appendix Table 3.

* Additionally, does it make sense to distinguish R_unvax, R_MCV1, and R_MCV2 in your compartmental model? Given that these compartments cannot become infectious perhaps a simplifying assumption would be to have just a single compartment for removed and examine the size of the flows into this compartment. The public health implication should be—but is likely limited by resources—that if it is possible to first determine antibodies, only those with no prior measles infection should be targeted for vaccination. I would suggest including the MSIR model equations in the main manuscript such that the reader knows the assumptions being made in the work

As mentioned previously, we tracked cohorts through age, space and time in our transmission model and all modeling equations have been included in the main text. We decided to track various recovered compartments across their vaccination statuses as it made for easy transformation and to track overall vaccination prevalence within the population and afford the ability to track which persons at any stage in our model have been vaccinated. The final results of our model are the exact same whether all infectious or successfully vaccinated persons have reached our differentiated R_unvax, R_MCV1, and R_MCV2 compartments or one final R compartment (i.e., the sum of the differentiated groups). Additionally, there is also utility in understanding what proportion of immunity is derived from RI or SIAs versus infection – given that the goal over time is for the proportion due to infection to decrease, then for reliance on SIAs to wane as RI coverage increases. This level of detail included in our model provides a more nuanced insight into the contribution of different vaccination approaches to current immunity patterns, and allows comparison of vaccination coverage, which is a commonly observed metric, and immunity in a unified framework.

Lines 202 and 231: This seems inconsistent with study period and age groupings provided previously

We ran our model for years 1980 to 2019, while fitting data from 2013 to 2019 (i.e., the years for which data was available). This is reflected in both sections describing the model as well as when the starting state was described. We have clarified the time period of fitting on line 275. With respect to age groups, we used 24 age groups (as described on lines 276-278, formerly line 202). However, during our starting state, we made the assumption that multiple of our 24 granular age groups had the same initial immunity and susceptibility profile, as we did not want to extrapolate from limited granular-age specific evidence on pre-vaccine susceptibility and waning maternal antibodies.

Line 209: Are other countries than Ethiopia being considered?

We thank the reviewer for pointing out this typo, as Ethiopia is the only country included in this manuscript. We have revised the following sentence (lines 282-283) to now read as:

“For each zone, we used a synthetic contact matrix standardized for zone population within each epiweek.”

Line 209: A comparison with on contacts in South West Shewa Zone of the Oromia Region by Trentini et al. (2021) [https://doi.org/10.1186/s12916-021-01967-w](https://urldefense.com/v3/__https:/doi.org/10.1186/s12916-021-01967-w__;!!K-Hz7m0Vt54!m2otYIa04JRryQhcbvL5e3sVNPBudWgzSNpDmAEcrq7PRDw71MR53uuvrt9v3PZ2ze3SOmc5ZDJJNQ$) would be nice

The contacts presented in Table 2 of the paper referenced by the reviewer seem to have little variation across age relative to the underlying age pattern of contacts derived in our manuscript from Prem *et al* (3) (<https://doi.org/10.1371/journal.pcbi.1005697>), which plausibly could be driven by different assumptions about contacts. Using this alternative contact survey would drive less social contact between especially adolescents and encourage more social transmission among young children relative to our current input data. Given what we know about social contact patterns from our Ethiopian co-authors, this referenced pattern of social contacts may less accurately reflect their interpretation of the social contact pattern within Ethiopia compared to the source we used.

Line 215: Suggest reference to Xia et al. (2004) [https://doi.org/10.1086/422341](https://urldefense.com/v3/__https:/doi.org/10.1086/422341__;!!K-Hz7m0Vt54!m2otYIa04JRryQhcbvL5e3sVNPBudWgzSNpDmAEcrq7PRDw71MR53uuvrt9v3PZ2ze3SOmcNzLzwZw$)

We have added this reference to now line 293.

Line 229: Would be better to include the MSIR compartmental model equations in the manuscript

As previously mentioned in an above comment, all compartment model equations have been added to the manuscript on lines 285-320 and lines 359-390.

Line 233: Unclear why this is relegated to supp. inf.

We have moved previous Appendix Figure 2 to the main text now as main Figure 2.

Line 238: “some” is vague, please quantify

This has been more extensively drawn out and expanded (lines 330-335) to now include additional details on our starting state including the following:

“All other persons were considered susceptible at our starting state apart from 4253 infected individuals to seed the infection. This was computed empirically by estimating infections in the first epiweek by taking the national, annual reported cases from 1980, dividing by number of epiweeks and applying an approximately 5% reporting rate. We distributed initial cases across age groups based on the age pattern observed from the earliest age-specific case notification data available (i.e., from 2013).”

Line 239: This is also vague, please qualify what exactly you tested and what “little to no difference” means

We have added this information by revising this sentence (lines 354-357) to now read as:

“We tested the sensitivity of these assumptions, and they yielded little to no difference in results (i.e., a difference of fewer than 100 estimated cases in the final year across zones), likely as the model had ample time to equilibrate since it was run for 33 years (i.e., 1749 epiweeks) before fitting to the first available data in 2013.”

Line 243: Should note that beta is the flow from the susceptible to infected compartment (assuming common notation used)

We have added this note to lines 359-360 to now read as:

“For each time step or epiweek ($i$), we computed the transmission probability, also known as the flow from the susceptible to infected compartments, ($\beta_{i}$) such that…”

Line 256: Provide context, is late March significant in Ethiopian culture or is this common to the measles virus?

We determined the seasonal peak of case notifications empirically from the underlying case data. Ethiopia does not to the authors’ knowledge have a significant cultural event in late March. There are many climatic zones in Ethiopia, including a temperate climate across many populated regions (such as Addis Ababa). Temperate climates suggest peak transmission for measles in the late winter / early spring, such as late March. Regardless, we chose not to make an assumption outside what was provided from the case data themselves, and to be more transparent to the readers have modified the following sentence (lines 372-374) to now read as:

“The seasonal displacement was calculated empirically by fitting a sine curve to reported case notifications and was determined to be approximately 2 (i.e., at the 13 week of each year).”

Line 272: Abbreviations already introduced not used

We have revised this sentence (lines 394-396) to include the previously mentioned abbreviations to now read as:

“Finally, based on already discussed RI and SIA MCV1 and MCV2 coverage values, we re-calibrate to weekly population-level vaccination prevalence.”

Line 275: Vague, please note which algorithms specifically

We have discussed which algorithms used for model fitting extensively in the Methods subsections called “Model fitting considerations: steep likelihood surface” and “Model fitting considerations: collinear parameters”, as well as described our final model fitting approach in subsection called “Final model fitting algorithm”. In this initial subsection, we were aiming to set the scene for our overall model structure without spending time initially discussing model fitting approaches. However, to guide readers to these later subsections, we have revised the following sentence (lines 398-401) to now read as:

“We tested several model fitting algorithms, including various Markov chain Monte Carlo (MCMC) samplers and block coordinate descent (described in detail below). For each, we estimated the following parameters: maximum and minimum transmission parameters over a season ($\beta_{max}$ and $\beta_{min}$) and a reporting rate ($\rho$).”

Line 279: Why was 5 chosen?

We have added the following additional information (lines 420-423) to describe how our dispersion parameter was selected:

“We tested various dispersion parameters (i.e., 1, 5, 10, 50) to improve likelihood acceptance in early modelling experiments. Increasing the dispersion parameter greater than 5 did not improve initial sampler acceptance rates, so therefore we used 5 for the remainder of experiments.”

Line 281: Inconsistent index for week, please see earlier suggestion on updating notation

We thank the reviewer for noting our error. This has been corrected and the week time index is now consistently represented as “$w$” as previously suggested by the reviewer.

Line 283: Please provide version for package used for reproducibility

We have modified this sentence (lines 423-424) to include package version, now reading as:

“We used R version 4.2.2 for this analysis and package Rcpp version 1.0.9 to build our transmission model.”

Line 287: No evidence is provided to support this statement, please add

We have updated this sentence (lines 428-431) to point readers to this evidence that can be observed in now Figure 3 across three small regions:

“As observed at the regional level (Figure 3, in Benshangul-Gumaz, Dire Dawa and Harari People regions), many zones reported cases only every other week, which is inconsistent with epidemiological expectations for measles transmission and may represent a data artefact generated in the reporting process.”

Line 292: Please examine this, what are the results of modelling fortnightly rather than weekly and how does it differ from your original results?

We describe using fortnightly cases rather than weekly cases based of an assumption we made about our data regarding whether the missing weeks truly are representing zero cases or if their cases are added to the subsequent week reported. This has implications for the strategy we have used to pre-process our data (i.e., testing fortnightly aggregations, but ultimately using a loess smoothing curve). Therefore, we are still modeling at the epiweek scale, but instead using data at a smoothed scale.

Line 302: Note which goodness-of-fit metric is being used

We have revised the following sentence (lines 453-456) to now read as:

“If these predicted transmission peaks fell in a separate month or quarter from the data, however, trivial differences in timing would result in poor model fits using likelihood-based fitting methods when all results were aggregated to the coarser time step.”

Line 308: LOESS should be capitalised as it is an abbreviation

We have revised the following sentences (lines 461-464) to now read as follows:

“Instead, we chose to smooth cases by zone and age group using a locally estimated scatterplot smoothing (LOESS) function with a span of 0.2. We tested LOESS functions with multiple spans for sensitivity, but ultimately chose 0.2 to maintain underlying variation across the time series and avoid over-smoothing.”

Line 315: Suggest adding the reference underlying this assumption here also (reference 30 cf. line 324)

We have additionally added this reference to the suggested line.

Line 318: Provide which “more recent years” are being referenced

This sentence (lines 473-481) has been revised to read as:

“However, this assumption yielded little to no transmission (Appendix Figure 2) in more recent years (i.e., after 2008) even with complete reporting due to high recorded vaccine coverage, which are both implausible as they do not match recent case reports.”

Line 320: Please provide the notation for this parameter so the reader can follow it

The notation $vax\_eff$ has been added to line 482 so readers can follow in the online code.

Line 332: Please compare with other methods such as hidden Markov chains and nowcasting

We agree with the reviewer that hidden Markov chains may be useful for this general type of question (i.e., inferring a latent state from an observed one). However, the general issue we grappled with (i.e., challenges in model fitting to many observations) would likely still extend to this framework. We would also likely face similar challenges in estimating collinear parameters such as vaccine effectiveness, case ascertainment rates and force of infection.

In addition, “nowcasting” – or the general premise of predictive modeling fitting to data without taking into account the underlying natural history – will also likely face similar issues. A nowcasting model of measles susceptibility would also likely need to have an autocorrelated feature with space and time as additional explanatory variables, so would probably have little explanatory power.

Line 335 and 340: Should this not be d rather than R (or d\in R) and is not this d for zone as previously implied in equation for force of infection?

The $d$ notation represents zones and, now updated, $Q$ regions (formerly $R$). Since the case data were being fit at the zone-level, we kept the left-hand side of both equations (i.e., $logit(\rho_{a,d,w})$ ) to be representative of what is being fit. The regional reporting rate (i.e., $\rho_{Q}$) is being applied to all zones within the region. We have clarified this by adding the following to the sentence on lines 498-501:

“To explore variations in reporting, we tested a model with region-specific reporting rates, $\rho_{Q},$ for each region $Q$, that is used across all zones $d$ in region $Q$ such that:

$logit(\rho_{a,d,w})=logit(\rho_{Q})$”

Line 357: Please provide a table with performance metrics

We provided tables with performance metrics in the Appendix (Tables 5 and 6). We have added reference to these tables later in the results section (lines 670-674) when discussing the final model selected:

“We fit bootstrapped samples using block coordinate descent across two different reporting structures (i.e., a single reporting rate and region-specific reporting rates) and four different vaccine effectiveness values; see Appendix Section 2 for full model results and the best model results per reporting structure including Appendix Tables 5 and 6 for model performance metrics across reporting structures.”

Line 365: Please provide details of computational environment as this can have an influence

We have provided R versions and all relevant package versions used during this manuscript (lines 423-424, 545, 577, and 582). Additionally, we added the following information to lines 424-425:

“We ran all computations in a distributed computing environment with 50GB memory and 4 cores.”

Line 367: Please provide stopping criteria and thresholds for algorithms used for reproducibility

We have added this information to our methods section on lines 607-611 to now read as:

“Across bootstraps, each block coordinate descent algorithm was run for 10 full iterations, regardless of if convergence had been met prior to this interval. Generally, convergence was reached before approximately 4 to 5 iterations (Appendix Figure 6) as evidenced by unchanging parameter results with further optimization.”

Line 376: Please provide package version and settings used for reproducibility

This has been updated (lines 544-545) to now read as:

“As such, we addressed this issue using a deterministic optimization of a derivative-free maximum likelihood estimator (via the *dfoptim* package version v2020.10-1).”

Line 377: Please quantify the fraction for comparison with previous estimate of 20 seconds

We have added additional context and an example in how long models took to finish and converge (i.e., approximately 5 hours) relative to MCMC models that would run for many hours (i.e., 72+) without approaching convergence. This has been added to lines 545-547 that read as:

“Models were able to successfully run while using a fraction of the computational resources (e.g., convergence in 5 hours relative to MCMC runs that failed to converge after 72 hours).”

Line 389: I don’t think this is sensitivity analysis, which consists of checking robustness of results

to assumptions made, but rather something like a profile likelihood two-stage estimation approach

We appreciate the reviewer’s suggestion and have revised this sentence (lines 563-566) to now read as:

“Instead, we decided to use a profile likelihood two-stage estimation approach across different vaccine effectiveness parameters to select the model with the best fit as determined by statistical criteria (i.e., Akaike Information Criterion [AIC] score).”

Line 401: This is inconsistent formatting with previous packages mentioned. Please provide a package version number

The package versions across all mentioned packages have been aligned (lines 423-424, 545, 577, and 582), including a package version number for all instances.

Line 402: Provide details of “initial starting state” for reproducibility

We have added details on parameter values for our starting state. The following (lines 579-580) have been added:

“Initial values parameters were as follows: $\beta_{max}$ = 0.25, $\beta_{min}$ = 0.12, and all reporting rates $\rho$ and $\rho_{Q}$ = invlogit(-5).”

Line 405: Provide convergence/optimisation settings

Convergence and optimization settings, with the exception of the number of maximum objective functions allowed to be evaluated in the second step transmission parameter estimation. We have noted this now on lines 581 and lines 590-591 that now read, respectfully, as:

“We used package default convergence and optimization settings.”

“We used default convergence and optimization settings except allowing 20,000 maximum objective functions to be evaluated.”

Line 409: MLE (as abbreviation is already introduced)

We have revised this sentence (lines 594-597) to now read as:

“We ultimately used MLE via block coordinate descent to first fit our reporting rate parameters (i.e., either $\rho$, or $\rho_{R}$ by region depending on the reporting structure for the model) while holding $\beta_{max}$ and $\beta_{min}$ constant, and then subsequently to fit $\beta_{max}$ and $\beta_{min}$ while holding our reporting rate parameter value(s) constant.”

Line 418: Is 100 samples enough? The data used is large and this number is small

We chose to run 100 bootstrapped samples leaving out 25% of the underlying data in each sample to generate simulated draws from a posterior distribution. During model fitting, we explored alternatively leaving out 10% of the underlying data, which produced more narrow uncertainty bounds for our parameters. While more samples could provide more underlying simulated draws to subsequently use for parameter uncertainty calculations or subsequent draw-level predictions, we do not believe the underlying mean values or range of uncertainty would be likely to subsequently change with increased bootstrap samples. Additionally, while our final models were more computationally feasible than various models tested, they still took over 5+ hours of run time; adding more samples would be increased and expensive computational demand.

Line 433: Figure 1 does not show zones. For comparison, standardised rates to account for

population size may make sense for Figure 1

To show readers the scale on which the case data are reported to highlight the reporting trends mentioned in our methods section, we chose to keep now Figure 3 on the scale of cases instead of a rate. Cases are represented as standardized rates in Figures 5 and 6 for comparison. Also, while regions are made up of various numbers of zones, we have decided to show now Figure 3 on the scale of regions to make the figure on a more easily interpretable scale with 11 panels (for regions) instead of 79 panels (for zones). Smoothed reported cases by zone are shown in Figure 7 and Appendix Figures 7-11 on scales of cases and also standardized rates.

Line 434: Please note that this is 364 weeks in total since that is the time unit being used (in addition to not instead of)

We have added the number of epiweeks (371) for reader context to this sentence (lines 631-633) to now read as:

“Twenty-one (of 79) zones reported fewer than 100 cases across the entire seven-year (371-epiweek) period, nine of which reported fewer than ten cases, and six of which reported no cases.”

Line 435: A graphical representation would help the reader

To add a graphical representation of the distribution of reported cases across zone-weeks, we have added Appendix Figure 4 (referenced on now line 633) which is as follows:

Line 442: Please provide intuition on which source is more likely to be correct. Corrections of time series for cases arising from different systems may have been considered during COVID-19 and may be something the authors could investigate

Within our authorship group, our co-authors with expertise within the immunization system in Ethiopia felt that it was not possible to determine which source of case notifications were more accurate. We have added a sentence (lines 643-644) noting this uncertainty in the methods section:

“It was unclear based on our knowledge of Ethiopian surveillance systems whether case data from subnational locations or national cases were more accurate.”

Line 449: Please explain why endemic transmission would not be expected in Ethiopia when you have previously explained that the second dose is only introduced a few years ago (at time of writing)

We agree with the reader that we would expect endemic transmission in Ethiopia. We recognize this previous description (lines 649-652) was potentially confusing and have revised to now read as follows:

“The observed age pattern of reported cases is unexpectedly that typically seen from countries or locations approaching measles elimination, not from locations with endemic transmission with relatively moderate vaccine coverage (such as Ethiopia).”

Line 467: This came out of nowhere, please introduce the socio-demographic index in the methods section

To introduce the socio-demographic index in the methods section, we have revised the following sentence (lines 615-618) to now read as:

“We used our posterior bootstrapped samples to compare regional reporting rates to regional metrics of socio-demographic index, which is a composite metric of fertility, education and lag-distributed income per capita, and to make predictions of measles incidence and susceptibility across age groups, zones, and weeks from 2013 to 2019.”

Line 471: I would suggest discussion of the distribution of remaining susceptible cases be the first part of the results section as that was the exposition given in the introduction

In response to suggestions from the other reviewers, we have re-structured and re-arranged some content to be focused generally on the methodologic considerations rather than a disproportionate focus on Ethiopia specifically; instead, Ethiopia has been re-framed as a case study example. As such, in our introduction, we have moved the description of Ethiopia to the end following the introduction of the operational and methods considerations. Therefore, the order of the results now matches the order of the introduction – discussing model fitting and then the practical example of Ethiopia to follow.

Line 478: Please quantify the “negligible differences”

This additional information has been added to now lines 691-701:

“When using a model with a single reporting rate compared to a regional reporting rate, there were negligible differences in susceptibility patterns across 0-to-4-year-olds (i.e., less than absolute differences of 1% in proportions), as approximately the same transmission parameters were estimated.”

Line 483: Please describe when, where, and which format the mass campaign events have for the unaware reader

We have added a table to our Supplementary Information (Table 2) to overview when campaigns in Ethiopia have occurred from 2000 to 2019. This table is now referenced on line 224 at the first mention of SIAs in Ethiopia.

Line 488: Should context be given that Somali region borders Somalia which is politically unstable?

We have added the following context to this sentence (lines 708-711) to now read as:

“In 2019 across 0-to-4-year-olds, zones in Somali region (i.e., the region bordering Somalia, which has recently experienced political instability) had the highest susceptible proportions compared to zones from other regions.”

Line 549: RI (abbreviation already introduced)

We have revised this sentence (lines 780-783) to now read as:

“One alternative option is that coverage from both RI or SIAs might not be as high as originally estimated or that there are limitations in the current available data, which are aggregated to zones, to capture true coverage heterogeneity.”

Line 570: Should context be given for the Gamo zone? Should this be zone? It is inconsistent with previous “region” in the sentence

Gamo is a zone (i.e., second-administrative unit) while Somali, which also experienced reported measles outbreaks in 2022, is a region (i.e., first-administrative unit. We have added the following sentence (lines 806-807) to give context to the Game zone:

“Gamo zone is one of the most populated yet rural zones within the country and located in the southwest.”

Line 577: What about displacement? This is a known driver as well as a known humanitarian health issue in Ethiopia

We have added displacement as an example, along with conflict, as a possible factor that may heighten disease risk in Ethiopia in years since 2019. This is now observed on lines 812 -814 and now read as:

“Future work will be needed to incorporate these effects as well as other factors that could decrease coverage or case reporting, increase susceptibility, and heighten disease risk in the years since the end of the study period (e.g., conflict, displacement).”

Line 584: But does demographic data exist on which to construct a synthetic contact matrix for these ages? Also, were they not included in the 1980 to 2019 window?

Demographic data exists in fine age bins (4), which we were able to use to construct our granular age bins used for our model. In our discussion, we wanted to highlight that while we can produce predictions at finer age bins, we are only fitting to data reported across coarser age bins, which may not allow us to fully understand age-specific dynamics compared to if we had single year or even more granular case-based data.

Line 597: The authors could also consider use of road maps

We have modified the following sentence (lines 838-841) to read as:

“These could include use of travel time metrics for persons traveling on foot, collecting data from road maps, or other model structures such as radiation models.”

Line 617: Rouge full stop

This has been corrected.

Line 619: Should presumably be [https://github.com/alyssasbarra/ethiopia_case_fitting](https://urldefense.com/v3/__https:/github.com/alyssasbarra/ethiopia_case_fitting__;!!K-Hz7m0Vt54!m2otYIa04JRryQhcbvL5e3sVNPBudWgzSNpDmAEcrq7PRDw71MR53uuvrt9v3PZ2ze3SOmdhGlD8Vw$)

The link the reviewer referenced is the code for a previous version of this manuscript used for an academic thesis submission. A new and permanent updated link will be provided at reviewer or editorial request or at time of publication.

Line 620: This is not possible to review as it is not available

These estimates are too large to add to the supplement; however, we will provide underlying estimates by zone and age upon request or in an online archive at time of publication.

Line 749: The authors should note in the caption that differing axes are being used

The following caption was generated for now Figure 3 (lines 1029-1030):

“Across regions, different y-axes are used to emphasize the underlying spatial and relative temporal pattern within each region.”

Line 776: The colours in the figure are difficult to distinguish in black and white, suggest use of different line types as well as annotating (could be geom_rect) where the study period is (2013 to 2019)

This figure (now main text Figure 4) has been revised as the reviewer suggested and the caption has been updated (lines 1050-1052) to read as:

“Figure 4. Reported annual suspected measles case notifications nationally (blue, solid line) from 2000 to 2019 via Joint Reporting Form and aggregated subnational case notifications to national scale (red, dotted line) from 2013 to 2019 during the study period (grey).”

Line 781: Discrepancy between reported suspected and reported in caption and axis label

The title and caption for now Figure 5 were revised (lines 1065-1066) to now read as:

“Figure 5. Reported measles incidence in Ethiopia in 2019 across reported five-year age groups.

Reported measles incidence from Ethiopia was generated from suspected measles cases. “

Line 793: Same comment as previously. The authors should note in the caption that differing axes are being used. The authors could consider adding vertical lines for the thresholds considered in the work (88 per cent and 47 per cent and so on)

The thresholds the reviewer notes were used to represent and test difference values of vaccine effectiveness, not vaccination coverage – which is what is shown in the figure. Therefore, highlighting these same values would be inappropriate as they have different underlying meanings. The title and caption for now Figure 6 have been revised (lines 1077-1082) to now read as:

“Figure 6. Reported measles incidence across regions in Ethiopia against first-dose measles-containing vaccine (MCV1) coverage for each zone-year among 0-to-4-year-olds from 2013 to 2019.

Reported measles incidence rate from Ethiopia was generated from suspected measles cases. Across regions, different y-axes are used to emphasize the underlying reported incidence rate within each region.”

Line 811: Suggest reversing the colour scale as most might expect a darker colour to be a greater value. The colours in the figure are difficult to distinguish in black and white and the borders are difficult to see for the darker colour

We have chosen to use accessible color palettes (provided via the R package “viridis” (5)) for color-blind persons for our figures where color differentiation is critical for understanding. The viridis package also coverages a range of brightness scales when desaturated. We are attempting to emphasize areas of high residual susceptibility and the viridis color scales are generally used in such a way as to have the brightest colors stand out. In order to maintain our accessibility and consistency with package and examples, we have left the color scales as. We apologize for any inconvenience this may cause.

Line 827: Please comment on the greater proportion susceptible found in 2019 and the public health implications of this

To comment on this finding and also add additional context to more recent outbreak reports already highlighted in our discussion section, we have added the following to lines 799-803 that read as:

“Our models suggested higher susceptibility broadly in 2019 relative to earlier years, likely stemming from overall low MCV1 coverage and a lack of recent vaccination campaigns with few recent widescale outbreaks. Given the unfolding situation in Ethiopia in years since 2019, these results suggest residual susceptibility gaps that leave many vulnerable children susceptible to preventable disease and death.”

Line 831: Please note points are sized according to population count

The caption for now Figure 11 (lines 1133) now reads as follows:

“Each point represents a zone, is coloured by region and is sized by population.”

Regarding the supporting information:
* As a general comment, it is unclear why the model and results have been relegated to the supporting information. As far as I am aware, PLOS Computational Biology does not have restrictions on number of figures in manuscripts, so it would make more sense to include them where they are discussed in the main text.

We thank the reviewer for the suggestion. The main model materials from the appendix have all been moved to the main text, which included previous Appendix Figure 1 (now main Figure 1) and previous Appendix Figure 2 (now main Figure 2). As previously mentioned, we have included all equations relevant to the main model to the main text (lines 285-320 and lines 359-390). An example of the result figure of fitted cases among 0-to-4-year-olds from the appendix (previously Appendix Figure 7) has been moved to the main text (now main Figure 7). The remainder are additional examples of model fits that can remain in the appendix for simplicity.

* LASSO should be capitalised as it is an abbreviation

This sentence (page 1) in the Supplemental Information has been revised to now read as:

“$\boldsymbol{X}_{\boldsymbol{s,t}}$ are the predicted surfaces from generalized additive models, least absolute shrinkage and selection operator (i.e., LASSO) models, and boosted regression tree models, which are space-time specific only (not age-specific).”

* Note package version for TMB package

We used TMB version 1.9.1, which has been added to the Supplemental Information (page 2).

* Suggest adding lines and labels to distinguish country (C_{t,g}, \logit(p_{t,g}), region (C_{t,c} etc.), and zone (C_Pt,a_1}). It seems there is a copy-paste error in the third binomial

Lines and labels have been added (page 2). Additionally, we thank the reviewer for noting our error. We have modified the third binomial equation appropriately.

* Which type of splines are being used?

The following has been added to page 3 of the Supplemental Information (page 3):

“Our generalized additive models use thin-plate regression splines on our covariates, modified to penalize null space slightly so that the arguments can be shrunk to zero, with starting k=3.”

* “Methods have been described elsewhere” is not sufficient

To provide additional context for the reader, the following lines on page 3 have been added to now read as:

“Models were calibrated (in logit space) to results from the Global Burden of Disease Study^2^; methods have been described elsewhere^1^. In short, to ensure the population-weighted national average of coverage from the subnational models were equivalent to national-level coverage estimates, the following calibration was used:

$$logit\left( V_{GBD,t} \right)=logit\left( V_{subnational, t} \right)+k_{t}$$

, where $V_{GBD,t}$ is national-level coverage for time $t$, $V_{subnational, t}$ is the population-weighted national average of coverage from subnational models at time $t$, and $k_{t}$ is a calibration factor for time $t$ that is chosen such that the un-logit-transformed means are equal after the subnational values are adjusted in logit space.”

* Should the campaign efficiency p not contain the risk for the opposite outcome in the denominator?

We have added the derivation of p to the Supplemental Information (page 4) that now reads as:

$$RR=\frac{p_{vax}|vaccinated}{p_{unvax}|vaccinated}$$

$$p_{vax}|vaccinated= RR* p_{unvax}|vaccinated$$

$$\# of doses administered=(p_{vax}|vaccinated*\# previously vaccinated)+(p_{vax}|unvaccinated*\# previously unvaccinated)$$

$$\# of doses administered= p_{vax}|vaccinated*\# previously vaccinated+ p$$

$$p=\frac{\# of doses administered}{\left( RR*\# previously vaccinated \right)+\# previously unvaccinated}$$

* Please describe how demographic change is incorporated

The demographic change is incorporated in the RI and SIA cohorting model by adjusting the size of each birth cohort in each year, as it is in the main transmission model presented in this manuscript. We have now noted this in the Appendix material (page 4) more directly to now read as:

“The cohorting model is run for each subnational unit through 2019 while also accounting for demographic changes (i.e., aging, births, mortality, and migration) using the same methods, assumptions and data inputs to those described for our transmission model (e.g., population surfaces from WorldPop calibrated to the Global Burden of Disease study).”

* All figures are difficult to distinguish in black and white

We have opted for color-blind, accessible color palettes for our figures where color differentiation is important for the interpretation of our figure. As previously mentioned, we have chosen to use a widely-available color scale that balances accessibility for colorblindness, perceptual uniformity, spanning a breath of colors to enhance the reader’s ability to detect smaller changes in the underlying metric (compared to, say, a sequential palette), and brightness.

* Please index figures by dates from 2013 to 2019 rather than numbers or provide a secondary x-axis with the date

All figures are now indexed by dates or additional information has been added to the caption to assist in translating numeric quarters or weeks to dates.

* Table A1: Page number are not included so suggest adding them or changing the heading to say “section”

The heading now reads as “Reported in section” instead of “Reported on page #”.

* Should the tables not say Ababa rather than Abeba?

An alternative spelling of “Addis Ababa” is “Addis Abeba”, which is the official spelling used by the Ethiopian Mapping Agency. All our figures and tables contain the alternative spelling of “Addis Abeba”, so for consistency we did not modify the naming for the tables in Supplemental Information.

* Table A2: Please emphasise where in the table the inconsistencies are to aid the reader (e.g. with bolded font)

Each row, representing each parameter, in now Appendix Table 4 (formerly Table 2) has inconsistent results suggested across each starting value “End” column. Therefore, we bolded each “End” column and noted this in the caption.

* Table A3: Please write log-likelihood for consistency wit following table

This has been modified in now Appendix Table 5 (formerly Table 3).

* Please comment on why there is absolutely no uncertainty in estimates in Table A4 (from row 4 onwards it is estimates of 1 with an interval of 1 to 1, which seems unreasonable). Is this indicative of a convergence problem?

The parameters in now Appendix Table 6 (formerly Table 4) were modeled in logit-space but presented in this table in proportion space to assist in interpreting on a scale that would be easier for reader comprehension. Therefore, there was variation in samples in logit-space but when converted back to regular-space all round to approximately 1.

* Figure 1A: What is appendix B section 1? Is there a second supporting information? If so please combine them rather than sending the reader back and forth between different documents

We thank the reviewer for noting this error. We have removed this reference from Appendix Figure 1, now main Figure 1.

* Figure A2: Please use dotted and dashed to avoid confusion. Please annotate the flows (currently most would be assumed to be the same without additional information given, e.g. subscripts) and provide the model equations

We have revised now main Figure 2 to now have dotted lines for vaccination transitions and dashed lines for loss of maternal immunity. As previously mentioned, since no differential equations were used in this model, all corresponding compartmental transitions are included in the main text information.

* Figure A3: it might be easier to distinguish model fit as a curve and observed cases as points. Why is this region specifically highlighted?

Allowing both the observed and estimated cases to be shown as lines highlights the overall trend between quarters and how, while the peaks and valleys of cases are misaligned, the trends are similar and seems to only lag by approximately a quarter. Bale zone was selected as an illustrative example; this has been added to the figure caption on Appendix page 15.

* Figures A4 and A5: Please improve the labels

We have updated the color legend labels for now Appendix Figure 3 and have added additional context on the panels in the caption for now Appendix Figure 3.

* Figure A5: Please note in the caption different axes are being used

The following (page 17) has been added to the caption for now Appendix Figure 3:

“For each parameter, different y-axes were used.”

* Figure A6: It is unclear what the added value of this figure is, the linearity could be mentioned in a sentence in the text

This figure (now Appendix Figure 5) was included so that readers could approximate R_0_ based on any fitted $\beta$ in the manuscript. R_0_ was calculated using a next generation matrix approach. Given that the figure may still have some value in being able to quickly assess the relationship between transmission probabilities and R_0_ values, we would prefer to retain it. However, the following sentence has been added to the methods section (lines 618-624) to describe this relationship:

“We calculated R_0_ using a next generation matrix approach (Appendix Figure 5) such that for each zone $d$ across age groups $a$ and $c$, we computed the following:

$$\delta_{a,c}=\beta*W_{a,c,2,d}$$

$$\tau_{d}=max(eigen\left( \delta\right))$$

, such that $\beta$ is a transmission probability and $\tau_{d}$ is the zone-specific R_0_ value. We took the mean $\tau_{d}$ across all zones to calculate the expected R_0_ from each possible $\beta$ value.”

* Figures A8, A9, A10, A11, A12, and A13: Please enlarge, these are too small to read or scrape. Consider using all the space on the page as well as allowing to go on separate pages

All figures (now main text Figure 7 and Appendix Figures 7-11)have been made larger and x-axes have been updated with years instead of weeks.

**Regarding the references:**
* Formatting of references is inconsistent with respect to use of ISO4 abbreviations and capitalisation of PLOS. I suggest authors harmonise their references

PLOS guidelines(6) for authors state they use the Vancouver style of references. We have used the referencing software EndNote in the Vancouver style to be consistent. However, we would be happy to take additional editorial guidance on reference formatting.

* No DOIs are given in the bibliography

PLOS guidelines(6) for authors also state that DOIs are optional in the bibliography. We would be happy to take additional guidance on reference formatting.

* Is Reference 19 different than Reference 4?

Yes, reference 4 outlines vaccination coverage estimation methods and results from the Global Burden of Disease study, while reference 19 describes the Global Burden of Disease Study overall, including information about the population sizes used for calibration.

* Unclear what “14th” in reference 30 means

We thank the reviewer for noting this error. It has been removed from the reference citation.

* For reference 39 why not link to arXiv itself rather than a Harvard page?

The Harvard page link has been removed. We apologize for missing this default setting from the web download to EndNote.

**Regarding PLOS Computational Biology specifically:**
* The manuscript does not currently adhere to the PLOS’s editorial and publishing policies ([https://journals.plos.org/ploscompbiol/s/editorial-and-publishing-policies](https://urldefense.com/v3/__https:/journals.plos.org/ploscompbiol/s/editorial-and-publishing-policies__;!!K-Hz7m0Vt54!m2otYIa04JRryQhcbvL5e3sVNPBudWgzSNpDmAEcrq7PRDw71MR53uuvrt9v3PZ2ze3SOmd-1OTYKw$)). In particular, the data availability is not fulfilled which is also the case for point 8 in their Table A1. As the data is aggregated by age group there should not be identifiability issues whereby the authors should release it such that other researchers can recreate/reproduce their work.

We appreciate the reviewer’s comment and understand the importance of transparency in reproducible research. Our case data used in this study was obtained through a Data Use Agreement with the WHO to protect the rights of the data shared and produced by colleagues in Ethiopia. Therefore, we do not have permission to share these data publicly as we do not own them. We would be happy to direct readers who might be interested in using these data first-hand to the data team at WHO that gave us access to these data to set up their own Data Use Agreements as appropriate and have added language (line 881) encouraging readers to reach out to the study team via email. All other datasets which are not proprietary have been made fully available. We hope that the reviewer and editorial team understand our limitations in being able to publicly release these case-based data.

* Regarding authorship, the authors should use the CRediT Taxonomogy as adopted by PLOS ([https://journals.plos.org/ploscompbiol/s/authorship](https://urldefense.com/v3/__https:/journals.plos.org/ploscompbiol/s/authorship__;!!K-Hz7m0Vt54!m2otYIa04JRryQhcbvL5e3sVNPBudWgzSNpDmAEcrq7PRDw71MR53uuvrt9v3PZ2ze3SOmfw3BNbIQ$)) which will provide transparency regarding contribution and suggests that R. Ramshaw, S. Rolfe, and J. Nguyen be upgraded from acknowledgement to author (based on the description of their contributions given in lines 624 to 626). Additionally, the authors could consider a different ordering of authors that allows the in-country researchers to take the prestigious first and last position cf. Nassiri-Ansari et al. (2024) [https://doi.org/10.37941/RR/2024/1](https://urldefense.com/v3/__https:/doi.org/10.37941/RR/2024/1__;!!K-Hz7m0Vt54!m2otYIa04JRryQhcbvL5e3sVNPBudWgzSNpDmAEcrq7PRDw71MR53uuvrt9v3PZ2ze3SOmcfHPx6yQ$)

Per the CRediT Taxonomy, we have added R. Ramshaw, S. Rolfe and J. Nguyen to the author list. Additionally, we appreciate the reviewer’s additional suggestion that would highlight the Ethiopian co-authors. Their expertise and insights have been invaluable to our research. However, the current first and senior author positions were indeed responsible for all appropriate typical duties of these authorship positions (i.e., first author developed all models, conducted all analyses, wrote the first draft of the manuscript and was responsible for all revisions; senior authors supervised the work, acquired funding sources and guided the overall project). Therefore, it would be dishonest to discredit the work by the current first and senior authors in favor or re-ordering the author list. We agree with the reviewer, however, that more country-led research projects in the field of infectious disease modeling and vaccination epidemiology are needed. Note that the current paper was intended to be mainly a methodological article around fitting epidemic models to country data, rather than about measles epidemiology or public health recommendations for Ethiopia. The Ethiopian co-authors were primarily epidemiologists and public health researchers; their contribution was vital for data interpretation and giving the context but they were not experts in mathematical modelling. For a paper with a more public health focus, we would strongly support in-country researchers taking either or both first and senior author positions.

Regarding the code repository:
* Contains no file suggesting which order to run code in, some are numbered and some not; adding a README file would improve usability greatly. Unit tests, documentation, and linting would also improve the code. As R is a functional programming language use of functions in conjunction with apply rather than loops should speed up the code for the R scripts.

We thank the reviewer for their suggestions. We have added a README file to the code repository that provides information on the general overview of the code files and how users may want to interact with them. The contents of this README are as below:

“Exploring the utility of subnational case notifications in fitting dynamic measles models in Ethiopia

**For data processing scripts, see the following files:**

- 01_prep_subnational_case_notifications.R
- 02_prep_subnational_births.R
- 03_prep_mortality.R
- 04_prep_migration.R
- 05_prep_subnational_population.R
- 06_prep_contact_matrices.R
- 07_prep_gravity_matrices.R
- 08_prep_coverage.R
- 09_save_loess_cases.R

To run models with single reporting rates, use script BCD_bootstrap_single_reporting.R, which calls the following Rcpp function scripts:

- Rcpp_BCD_rho_fitting_bootstrap_district_single_age.cpp
- Rcpp_rolling_average_estimated_no_eff_subnat_only_down_adjust_regional_reporting_vax_eff_vary2_loess_seasonal_serial_interval_incidence_autocorrelation_nbinom5_single_reporting_bootstrap_district.cpp

To run models with regional reporting rates, use script BCD_bootstrap_regional_reporting.R, which calls the following Rcpp function scripts:

- Rcpp_BCD_rho_fitting_bootstrap_district_regional_age.cpp
- Rcpp_rolling_average_estimated_no_eff_subnat_only_down_adjust_regional_reporting_vax_eff_vary2_loess_seasonal_serial_interval_incidence_autocorrelation_nbinom5_regional_reporting_bootstrap_district.cpp

The remaining files are used for diagnostics for boostrapped samples (diagnostics_bootstrap.R) and making predictions and overall diganostic plots (predict_and_diagnostics_overall.R).”

* Note that certain auxiliary functions seem to be out of limits in the code repository, scripts such as “setup.R”, “misc_vaccine_functions.R” and “get_population.R” are not provided meaning other users may not be able to use the code as provided. I would suggest the use of a container to ensure correct versions of packages are used (as R has known backwards compatibility issues) or at least noting them in the manuscript.

We thank the reviewer for noting these functions to be missing from our code repository. We have updated the repository to include the underlying code mentioned. This code was run on high performance computing resources maintained by the University of Washington / Institute for Health Metrics and Evaluation. Due to security protections, file paths where these data and additional reference code had to be removed; these file paths would need to be modified to match the user’s computing environment in order to re-run the code, not that this precludes reproducibility. However, we apologize for any inconveniences this may cause.

* Assuming use of ggplot, if the authors wish to ensure their plots all have the same look (e.g. figures A4 and A5 in the supp. inf. look different), they could consider use of theme_set()

We thank the reviewer for their suggestion. As all main figures have matching aesthetics, we have left the supplemental themes the same and will consider for future research.

Reviewer #2:

Recent years have seen a mass resurgence of measles throughout the world with low and middle income countries (LMICs) being hit particularly hard in terms of morbidity and mortality. Measles susceptibility reconstruction, namely the attempt to estimate latent (typically age and space structured) susceptibility of a population to measles infection, is therefore a very timely topic of substantial relevance to both modelling and operational communities.

Assessment of susceptibility, however, is nontrivial and there is an interest in exploring potential new approaches to improve reconstruction. The authors contribute an additional modelling approach that complements other work already being done in the broader modelling community. Their approach attempts to leverage a substantial amount of data (notably suspected case data, vaccine coverage data, and population data) in hopes of explicitly quantifying susceptibility.

Generally, the paper is methodologically interesting and raises good points about some of the challenges of highly parameterized models in the face of extreme data frailty. Unfortunately, the overall goal of the paper is unclear. It is difficult to tell if the paper aims to be a discussion of methods and challenges (which would be interesting and valuable) or if it aims to present the results and (presumably operational) recommendations pursuant to this particular implementation. If the goal is the latter, the authors may need to dramatically expand the evaluation and operational interpretation of the model. In either case, the paper would benefit from having a clearer stance on what it is trying to accomplish.

If the authors opt for a methods focus, I think the paper (with a bit more information and discussion placing their model into context with other similar methods) could be a valuable contribution. Particularly as many groups may be interested in pursuing direct quantification methods for susceptibility reconstruction (as opposed to prioritisation oriented methods) it would be useful to have a publication highlighting the challenges such an approach may face.

We thank the reviewer for their thoughtful comments and feedback. The reviewer shares similar feedback to another reviewer about streamlining the messaging and goals of our manuscript. We have done our best to re-focus our manuscript to be centered around the data and model fitting challenges, while using Ethiopia as a case study. As such, we have revised the following sections:

- Abstract (lines 64-69): to highlight the methodologic changes and mention Ethiopia as a case study
- Author statement (lines 94-104): Re-focusing the parts regarding Ethiopia to be framed as a case study
- Introduction (mainly lines 212-229): moving content about Ethiopia out of the forefront, to set up the problem statement (i.e., data and statistical challenges) and then introduce our case study
- Methods (lines 257-266): adding additional information on methods used to estimate vaccination coverage to the main text to add methodologic details

The reviewer notes multiple points below regarding further discussion of our methodologic considerations in the broader context; please see specific responses to these comments below.

**Particularly positive aspects:**
1. The methods explanation, while dense, is very detailed and I greatly appreciated the transparent discussion of challenges the team faced in fitting as well as the very complete discussion of what strategies were tested (as opposed to only reporting the final method used).

We thank the reviewer for their positive feedback. We agree that discussing the challenges faced when fitting models and the complexity around using large, noisy datasets largely gets ignored across our field and is a valuable contribution to spark conversation between researchers about how to face these ongoing methodologic considerations.

2. The conversation of computational cost is an important one (particularly for resource constrained settings) an it is interesting to see methods that explore ways to leverage bootstrapping to create deterministic (and therefore computationally cheap) methods that retain a notion of uncertainty; while this uncertainty is somewhat "constructed", this is an issue that the authors clearly acknowledge and I do not see an apparent better solution than what they have proposed.

We appreciate the reviewer support in our attempt to consider computational demand and feasibility in our model fitting process. We agree with the reviewer that for in-country research teams across resource-constrained settings, access to complex computational machinery and resources (e.g., distributed computing environments) may not be acquired in the short or medium term. Discussing what models can be fit in the absence of these intricate infrastructures should be prioritized among researchers in the global health space to promote equity and encourage research to occur within countries themselves. Based on other reviewer comments, we have also added details about our computational environment throughout the manuscript that will provide additional context to these considerations.

3. Generally the discussion of limitations surrounding mobility modelling, uncertainty, and certain data frailty issues is thoughtful and well appreciated.

We thank the reviewer for the opportunity to explore complex and interconnected issues and how they relate to measles modeling exercises.

4. Great to see a large number of collaborators/authors from the study country (here Ethiopia) on the paper !

We have greatly valued the expertise and deep context-specific knowledge of our Ethiopian colleagues and are extremely appreciative of their contributions to this manuscript.

5. Glad to see the commitment to open source code.

We thank the reviewer for their support and agree that code should be open source to create a transparent and collaborative environment of research.

**A more neutral point:**
1. Case ascertainment of 3%. While we know that measles is massively underreported, 3% ascertainment is very concerning (though this is not to say the estimate is necessarily unrealistic). It would be fascinating to see other work looking into periods of active followup (though I am not aware of anything published for Ethiopia) or an analysis of potential reporting shifts seen during a period of active case finding (for example during a riposte) if there are any in the historic data.

We agree with the reviewer that a 3% case ascertainment rate is very concerning. Although we have searched for and are also unaware of any investigation in Ethiopia specifically that would allow us to validate this result, we have referenced this value to other estimates of measles burden – such as from modeled estimates produced by the World Health Organization(7). These results suggest an inferred global case ascertainment rate of approximately 2% (i.e., 205,173 cases reported / 9,232,300 estimated cases).

**Some things to consider:**
1. Data sources. It is not clear where a lot of this data comes from and it is therefore difficult to assess its associated reliability. For example, MCV1 coverage estimates can vary dramatically depending on where you get them from. It would be interesting to know if the authors are using administrative/independent estimates and if they compared multiple sources of coverage data to investigate quality. Similarly, it would have been nice to see a check of whether the coverage negatively correlated with epidemic risk (as would be expected). While I very much appreciate that the authors take time to discuss data quality (particularly for case data), a bit more nuance would be helpful. For example, the authors note that they don't see the expected negative correlation between suspected cases and vaccine coverage and conclude that this likely speaks to the poor quality of case data. It is equally possible, however this result has more to do with issues of vaccine effectiveness and/or issues in the coverage data (which can sometimes rely on highly frail and inaccurate population estimates).

We thank the reviewer for the opportunity to provide more information on the data used in our model. Estimates of vaccination coverage have been generated primarily from household-based survey data and model-based geostatistical techniques that rely on the assumption that locations closer in space and time have more similar coverage. We also considered doses reported to be administered through campaigns to construct RI and SIA coverage. To add more information in the main text for readers, we have added the following on lines 257-266 on data sources and methods used for estimates of vaccination coverage:

“In short, household-based survey data on MCV1 and MCV2 coverage are geolocated to the most geographically granular level possible. For RI MCV1 coverage, we used a model-based geostatistical framework to estimate coverage under the assumption that coverage is more similar in years and locations that have similar covariate patterns and in years, locations and age groups that are closer across time, space and age. For RI MCV2 coverage, less data was available to include in models, as MCV2 was only introduced in Ethiopia in 2019. Therefore, we used a hierarchical model at the zone level. For combined RI and SIA coverage, we developed a model tracking cohorts through time, accounting for RI coverage increases along with doses reported to be administered during SIAs.”

We appreciate the reviewers comment that the apparent discrepancy while comparing the reported case data to vaccination coverage may be in part due to errors in enumerating coverage or with considerations related to vaccine effectiveness we later consider in our manuscript in more depth. We have revised our interpretation (lines 656-659) of now Figure 6 displaying this relationship, to now reads as:

“The lack of negative correlation between reported incidence and vaccine coverage suggests variability in case reporting, inaccuracies in estimates of vaccine coverage such as errors in the numerator and/or denominator), variability in vaccine effectiveness, or some combination of these factors.”

2. Operational purpose. The authors suggest that this model will somehow be used to inform SIAs, though how that would be done and why explicit quantification of susceptibility (as opposed to prioritisation) is required is unclear. Explicit quantification is challenging and providing an explanation of why this may be worth the additional modelling effort over a ranking/risk oriented model would be useful. If, following the challenges faced, the authors conclude that perhaps a prioritisation approach would have been equally good or better, this should also be discussed.

While the model is not explicitly being used to inform decision-making (and we do not claim this), models like this could be used to inform subnationally or age-targeted SIAs by providing a snapshot of immunity profiles across age and space that could then be used for prioritization. By first providing a quantitative metric of susceptibility (either by counts of susceptible people or as proportions), decision-makers can use whichever is most relevant to the questions at hand – whereas prioritization exercises may lack granularity that can be useful in making nuanced decisions. Despite these benefits, the challenges faced during these exercises were non-trivial—and in specific settings and contexts may be too great to overcome. Therefore, we see this approach, if possible, to be another tool to be used in tandem with other resources, such as vaccination coverage or prioritization / risk-based models. We discuss this further as it very closely relates to the reviewers following point 7 on broader operational considerations.

3. On SIAs. The authors do not seem to have a perfectly stable definition of who SIAs should target, line 119 suggests SIAs should target areas with moderate coverage but 136 indicates that targeted interventions should look for vulnerable (unprotected) persons. It is also worth noting that claim of line 119 is highly debatable and ignores the fact that SIAs and other targeted interventions in LMICs will often face resource constraints limiting the number of people vaccinated. In these cases, one might elect to target certain priority groups, often those facing highest risk of mortality (children <5), even when a broader age target may do a better job at reducing overall transmission.

We thank the reviewer for pointing out this inconsistency in our manuscript and the opportunity to clarify. We agree with the reviewer that our previous phrasing could be interpreted incorrectly and ignore important operational considerations for those planning SIAs and other targeted interventions – and how these interventions should be prioritized over broad routine immunization system strengthening.

We wanted to highlight where models or estimates of susceptibility across age or geography might be operationally useful in decision making around SIAs or other interventions. While settings with low overall coverage are a priority for intervention, these countries might not benefit from highly geographically tailored interventions and instead rather general improvements in routine immunization outreach or even nationally-targeted (or broad age group targeted) SIAs or interventions or campaigns. Alternatively, countries with very high coverage will also likely not benefit as much from geographically or age-targeted campaigns as susceptibility gaps are likely more finely distributed across extremely local or social networks.

To better capture this nuanced discussion, we have revised now lines 120-129 to read as:

“Targeted interventions might be most operationally useful in countries with moderate vaccine coverage, as targeting approaches by age or geography might not be as relevant in countries with low overall coverage (where all areas or subgroups need coverage improvements via, for example, nationally-targeted SIAs). Alternatively, high coverage settings with pockets of persistent transmission among communities without explicit geographic division (e.g., among specific social networks), might need different strategies, such as tailored communication efforts and discussions of the risks and benefits of vaccination, to increase coverage beyond the use of geographically or age-targeted campaigns. A similar motivation in moderate coverage settings could also exist for implementing routine immunization strengthening efforts by location.”

Additionally, to be more consistent, we have revised now lines 160-163 to read as:

“Nevertheless, operational decisions need to be made based on practical and logistical considerations alongside various data streams that identify communities likely to most benefit from targeted interventions to prevent avoidable measles infections in short- and medium-term time horizons.

4. Complexity. The proposed model is quite complex, requiring several assumptions and facing potential problems of parameter identifiability pursuant issues of multicollinearity. While similar challenges likely face other modelling approaches, some of the assumptions may warrant some additional justification (for example, theta is set at 0.99 with no explanation) and or at minimum some discussion. For example, the authors choose to bring vaccine effectiveness into their model, rightfully noting that it is subject to being undercut by issues of cold chain breakdown and/or high prevalence of malnutrition. Cold chain problems and malnutrition, however, are often characterized by substantial spatial heterogeneity, which should presumably then be reflected in the model but does not seem to be. It would also be interesting to see more discussion of whether the authors believe all assumptions were ultimately necessary for adequate model performance. It may also be interesting to consider the degree to which the model complexity (in addition to the artificial certainty of the data, as noted by the authors) may have contributed to the steepness of the likelihood surface.

The reviewer’s valuable feedback highlights a significant tradeoff that we were constantly considering while developing this model – the balance between model complexity (and perhaps accuracy of real-world processes and dynamic environments) and simplifying assumptions (and often as a results more straight-forward statistical problems). This tradeoff, as the reviewer notes, is not specific or unique to our model, but rather quantitative exercises more broadly.

To add additional context, we have clarified the following assumptions by editing the related text:

- Lines 319-320: “To aid in model identifiability, we assume $\psi$ to be 0.99 based on in-country expertise.”
  - Additional context for the reviewer: We selected this parameter value based off conversions with our co-authors from Ethiopia, where the consensus was that a vast majority of children – who measles mostly affects – do not travel outside their home zone on a weekly basis. We explored fitting this parameter, however it was computationally intractable in our current modelling framework.
- Lines 388-390: “$\alpha$ is a parameter, assumed to be 0.99 for purposes of model identifiability, to account for mixing parameters of the contact process or the discretization of a continuous process.”

The reviewer notes possibly changing vaccine effectiveness by geography related to variation in cold-chain problems and malnutrition; we agree with the reviewer that this is extremely plausible, though we assumed a single national vaccine effectiveness parameter. This is an example, of many, simplifying assumptions necessary for the model to be identifiable and fit. Assuming a spatially varying vaccine effectiveness parameter and a spatially varying reporting rate would be extremely challenging without additional data to ground our model, as changes in one parameter could directly affect the other (i.e., high vaccine effectiveness would also likely suggest less susceptibility, less estimated infections, and therefore higher case reporting). While we are interested in exploring these inter-connected, interesting and nuanced points, this is a limitation we are not able to currently address without additional data. To discuss further, we have added a limitation (lines 845- 850) to our discussion section:

“Also, although we assumed a single national vaccine effectiveness parameter, it is possible that vaccine effectiveness is heterogeneous across space, related to subnational variation in cold-chain problems and malnutrition, among other factors. However, given the challenges in fitting the model described, additional data would likely be needed – or other parameters would need to be assumed – to allow for a model that captures this additional level of detail to be identifiable.”

5. Issues of generalizability. It is unclear if this model is expected to be generalizable to other settings and what adjustments would be needed to adapt it (for example in the assumptions and fixed parameters like vaccine effectiveness). If the goal is operational recommendation/impact, a discussion of generalizability is warranted.

The overall model presented has been written to be generalizable to other settings, and with adequate input data and expertise from in-country partners could be used to generate similar results across other settings. Regardless of whether this specific model is used for these purposes in future research or operational efforts, the main themes discussed in this manuscript are certainly generalizable to other settings with similar surveillance systems likely facing similar data-related and computational challenges.

We have added the following to lines 727-734 of our discussion to capture our intentions on the generalizability of this work to other locations:

“While we used the example of Ethiopia as a case study, we expect the methodological considerations, challenges faced both in data interpretation and model fitting, and overall discussion on these topics to be widely generalizable to other settings with similar surveillance systems and data availability. With appropriate and similar input data as used for Ethiopia in this work and in-country expertise to guide parameter assumptions (e.g., a range of plausible reporting rates, vaccine effectiveness values, probability of staying in home subnational unit in a given week), our model could easily be applied to other locations.”

6. Model validation. While it is nice to see the use of the AIC for model comparison, it would have been valuable to see some work done on model validation, particularly if the goal is operational impact. The authors do this very slightly in their discussion of increasing trends in the Somali region and Gamo zone, but more work is warranted to give a proper explanation of whether the susceptibility estimates of the model make sense in light of real world trends. While the authors correctly raise concerns about the quality of suspected case data, which may make it difficult to interpret correlations (or not) between the estimates and case numbers, they could still for example, look at comparing with something like general epidemic risk. Epidemic risk will of course rely on case data, but should be meaningfully more robust.

While we did build our model iteratively guided by comparisons to the underlying case data, we agree it would be optimal to be able to validate the results of our model in the context of real-world trends; this is also noted by another reviewer. These could be through a comparison of seroprevalence estimates in recent years to model generated estimates of susceptibility; however, the only seroprevalence data in Ethiopia that is available is from 1994 and 1999 – both of which in the period before subnational case data were available. Therefore, despite the time lag, we have chosen to compare our results from 2019 to more recent outbreak reports from 2022. This is challenging to disentangle, though, due to possible changes in subnational measles epidemiology that unfolded within Ethiopia over the evolving time as well as large disruptive events such as SARS-CoV-2 pandemic, conflict, and related displacement. Also to note, these outbreak and case reports from recent years are also likely subject to similar quality- and completeness-related concerns as the data used to fit our models. As additional data sources to compare model results may be collected or released, we are keen to continue to validate our model and related outputs. We have added a limitation to our discussion (lines 832-834) to note the challenges on validating this model to real-world trends that reads as:

“Additionally, in the absence of recent seroprevalence data or alternative data sources, it was challenging to validate the results our modelled results in Ethiopia to recent real-world trends.”

7. Broader operational considerations. If the goal of models like this is to inform operational decision making, some consideration should be given to the anticipated challenges/feasibility of real world implementation. For example, is it realistic to implement such a model in an LMIC long term? Is the model worth implementing over a less complex model that can only provide a prioritisation output? Or, even further, since model susceptibility estimates correlated with vaccine coverage estimates, it would be good to discuss why operational decision makers should use this model over a strategy that simply chooses to target age groups / areas with the lowest estimated coverage (which is what many countries currently do).

We appreciate the reviewers feedback and note the complexity around using these models to inform operational decision making. The benefit to a model that provides direct quantification over prioritization is that decision-makers can toggle between exploring how prioritization would be made based on the relative number of susceptible persons by age or geography (i.e., most direct impact) versus the proportion of persons susceptible by age or geography (i.e., a more equitable, but perhaps more costly, approach). These methods also give numbers of children who need to be reached, which could help to inform the scale on which operations need to be performed.

However, complex models are often challenging to communicate to decision-makers and come with many limitations that hinder their ability to be directly applicable to real-world scenarios. For these models to be most useful, they would need to be frequently updated with the most recent data on vaccine coverage and case notifications in as close to real-time as possible. We know in many settings this would be extremely difficult; one of our goals of this manuscript was to illuminate some of these challenges.

In light of these limitations, we believe that ultimate goal of these kinds of models should not be to replace existing data sources used for and methods/mechanisms to make operational decisions (such as the WHO rule of thumb), but rather an additional tool that decision-makers can use to inform their operations along with more traditional estimates like vaccination coverage. The reviewer notes the correlation we observed between coverage values and susceptibility estimates; in this instance, decision-makers might be able to use these results and have more confidence in prioritization based on coverage knowing alternative results suggest similar findings.

To explore these considerations further with readers, we have added the following to our discussion section (lines 868-871):

“Given these limitations broadly along with current operational practices (i.e., making prioritization decisions based on triangulating estimates of vaccination coverage), modelled results of this kind are best suited to be used alongside existing processes and data streams as an additional resource for decision-makers."

8. Rubella. No discussion is given to how case misclassification (particularly with rubella) may have impacted the outcome of the model. It would be interesting to see a discussion of whether the model is equally generalizable to settings with high/low rubella burden, particularly in areas using the monoantigen measles vaccine.

We thank the reviewer for noting rubella, or other diseases that could cause case misclassification. While measles cases on the population-level are greatly under-reported, cases at the individual-level are often over-reported (i.e., misclassified as measles when they are in fact not). While the underlying susceptibility results could be impacted by not adjusting for any test-positivity rate, we believe that regardless of underlying rubella burden in a setting, the main findings and challenges identified in our work are still applicable and relevant to consider. However, we have added to the existing limitation on not considering test positivity rates in our discussion section (lines 819-822) to further discuss this shortcoming of our work:

“We only considered suspected measles cases and did not consider lab-confirmed measles cases or adjust for test positivity rates. Therefore, the cases we considered in this model may have been misclassified as measles instead of, for example, rubella.”

One other small issue, the authors state they have used R version 5.4.0, which is presumably a typo for 4.4.0 (the latest current version as of March 2024).

We thank the reviewer for noting our error. We have revised the following sentence (lines 423-424) for accuracy:

“We used R version 4.2.2 for this analysis and package Rcpp version 1.0.9 to build our transmission model.”

Reviewer #3:

This article considers a valuable, yet challenging, issue, of identifying subnational areas of measles susceptibility in high-burden settings. Results from these models could be highly beneficial to plan impactful vaccination campaigns and reduce preventable measles burden. Overall, I found the manuscript to be very promising: as the authors note, there are a number of challenges to navigate to design and fit such models. I felt that the authors made useful contributions presenting and discussing these challenges and how to overcome them.

However, on first-reading of the manuscript, I found the positioning of the paper inconsistent between sections: it unclear whether it was aiming to be a methods paper (focussing on the methodological contributions and challenges of the approach in general) or a paper about the trends in subnational measles susceptibility in Ethiopia. My main suggestion for improvement would be to focus the paper on one of these only: given the significant effort made on overcoming the methodological challenges, I would suggest focussing on this part, using measles in Ethiopia as a case study / motivating example, thereby bringing the best parts of the research to the forefront; I have tried to make specific comments to that effect below.

We thank the reviewer for their valuable feedback and appreciate the opportunity to strengthen our manuscript based on their comments. As the reviewer has suggested to focus on methodological challenges using Ethiopia as a case study, we have refocused various sections and have made modifications to bring the main theme to the forefront of our manuscript. Please see below for responses to specific comments regarding these modifications.

**Main comments**

1. Abstract / authors’ summary / introduction. I felt each of these seemed to be telling a different story, rather than all setting the scene for the same paper. Can the authors make changes so that these three sections are more cohesive, e.g. mentioning the data and model fitting challenges (currently only in the authors’ summary).

We thank the reviewer for the opportunity to improve our manuscript by streamlining our focus. We have revised our abstract to be more in line with re-focusing to center on challenges related to case fitting and using Ethiopia as a case study. Our abstract (lines 52-91) now reads as follows:

“In many settings, ongoing measles transmission is maintained due to pockets of un- or under-vaccinated individuals even if the critical vaccination threshold is reached nationwide. Therefore, assessing the underlying gaps in measles susceptibility within a population is essential for vaccination programs and measles control efforts. Recently, there have been increased efforts to use geospatial and small area methods to estimate subnational measles vaccination coverage in high-burden settings, such as in Ethiopia. However, the distribution of remaining susceptible individuals, either unvaccinated or having never previously been infected, across age groups and subnational geographies is unknown. In this study, we developed a dynamic transmission model that incorporates geospatial estimates of routine measles vaccination coverage, available data on supplemental immunization activities, and reported cases to estimate measles incidence and susceptibility across time, age, and space. We developed a time-varying compartmental model to estimate age-specific measles incidence and susceptibility. We use gridded population estimates and subnational estimates of routine and supplemental measles vaccination coverage. To account for mixing between age-groups, we used a synthetic contact matrix, and travel times via a friction surface were used in a modified gravity model to account for spatial movement. We explored model fitting using Ethiopia as a case study. To address data-related and statistical challenges, we investigated a range of model parameterization and possible fitting algorithms. The approach with the best performance was a model fitted to case notifications adjusted for case ascertainment by using maximum likelihood estimation with block coordinate descent. This strategy was chosen because many data observations (and likely presence of unquantified uncertainty) yielded a steep likelihood surface, which was challenging to fit using Bayesian approaches. We ran sensitivity analyses to explore variations in vaccine effectiveness and compared patterns of susceptibility across space, time, and age. Substantial heterogeneity in reported measles cases as well as susceptibility persists across ages and second-administrative units. These methods and estimates could contribute towards tailored subnational and local planning to reduce preventable measles burden. However, computational and data challenges would need to be addressed for these methods to be applied on a large scale.”

Additionally, we have made small modifications to our Author Statement (lines 94-112) to be in alignment with our re-focused objective. The statement now reads as:

“Estimates of subnational measles susceptibility are critical for planning targeted immunization interventions. In this study, we used subnational case notifications available from Ethiopia from 2013 to 2019 as a case study to fit dynamic transmission models of measles with various reporting structures. After exploring biases inherent in these case data, we used various model fitting approaches to consider how best to include these case data in transmission models. Following our investigations, we used a deterministic optimization algorithm via block coordinate descent to fit bootstrapped models with different reporting structures (i.e., single reporting rate and region-specific reporting rates) and conducted a sensitivity analysis across multiple vaccine effectiveness values. We discussed various considerations that need to be made when fitting dynamic transmission models broadly to subnational case notification data based on their inherent biases. These include:

- accounting for sporadic temporal case reporting,
- fitting models to biased and variable case notifications despite their certainty based on statistical calculations,
- considering how best to estimate parameters that may be collinear (i.e., transmission probabilities and reporting rates),
- accounting for various reporting mechanisms and how they may contribute to under-reporting, and
- exploring implications related to assumptions on vaccine effectiveness.”

The reviewer suggests below in their point 2 about re-structuring the Introduction, which we do as they suggest below.

2. Introduction.
   a. I felt that this focussed too much and too early on Ethiopia, when in fact little of the paper was focussed on Ethiopia specifically. I would suggest that the authors restructure the introduction to introduce first the problem (subnational measles susceptibility estimation) and application (targeted vaccination), then the challenges (case reporting patterns and model fitting difficulties, etc.) and finally present Ethiopia as a case example.

We have restructured our introduction section based on reviewer feedback to now focus on the methodologic considerations and then highlight Ethiopia as a case study or example. This largely involved moving the introduction to Ethiopian vaccination coverage, campaigns, and case notification data to now lines 212-229 (i.e., the end of the introduction section).

b. To highlight that this question/model is relevant more broadly, can you also add 1-2 sentences on other settings where this would be relevant.

We have modified the end of our introduction section (lines 203-210) to now read as follows:

“In this work, we explored methodologic considerations that would be necessary when using case notification data in transmission models to understand measles dynamics and estimate susceptibility by age, location, and time, and examined limits of data quality and implied underreporting rates. We used Ethiopia as an example of settings with these types of available case and vaccination coverage data. However our investigations and findings are broadly applicable to other settings with similar data and epidemiologic questions related to measles susceptibility and vaccination program planning.”

3. Methods.
   a. Again, I would suggest that the authors restructure to present the general model first, then the data and model fitting considerations (below).

We currently present the main underpinnings of our transmission model in our methods section from lines 272-425, then went on further to discuss the considerations made relative to our baseline model that required modification to make sense of the data available (lines 427-501) or for statistical convergence (lines 503-626). As these sections build on one another, we have kept the narrative order for the cohesion of this section. We have aimed to balance the reviewer’s suggestion and those of the other reviewers in making modifications to the methods section. We would be happy to take additional editorial guidance in this area.

b. Some of the “Model fitting considerations” are more considerations about the data. I think it would be clearer if the authors split the “Model fitting considerations” into two: one on the data (reporting patterns etc.) and one on the model fitting proper (collinearity, steep likelihood surface).

We have changed the headings on the following sections to be clearer they are related to how we considered modelling related to data challenges and limitations compared to statistical complications. Topics now have been modified to have the following headings:

- Data considerations for model fitting: sporadic case reporting (line 427); vaccine effectiveness (line 469); variation in reporting rates (line 487)

- Statistical considerations for fitting: steep likelihood surface (line 503); collinear parameters (line 549)

4. How can results from this model be validated/evaluated? There are some ideas when discussing the results from the Ethiopia data (comparing model-estimated susceptibility to percentage unvaccinated (1 - MCV1)), but I think it might be useful to draw these out more explicitly. Additionally, are there any other metrics the authors could use to validate part or all of the results, and what (if any) are the limitations or biases of these? For example, can the authors compare their estimates to the serosurvey data?

While we did build our model iteratively guided by comparisons to the underlying case data, we agree with the reviewer that it would be optimal to be able to validate the results of our model in the context of real-world trends; this is also noted by another reviewer. On lines 803-812, we include a comparison of our 2019 results to more recent reports of outbreaks from 2022. However, differences in results are challenging to interpret due to changing subnational measles epidemiology that unfolded within Ethiopia over time as well as large disruptive events such as SARS-CoV-2 pandemic, conflict, and related displacement. Also to note, these outbreak and case reports from recent years are also likely subject to similar quality- and completeness-related concerns as the data used to fit our models.

Because vaccination coverage values alone are not comparable to susceptibility estimates that include a lack of vaccination, loss of maternal immunity and a no measles infection history, a comparison between these two sources would be interesting for considering how different operational decisions could be made based on the varying estimates; however, a direct comparison to evaluate the model would be inappropriate as they are measuring different things, and the vaccination coverage data are inherently used as inputs to the modeled susceptibility estimates.

The reviewer notes comparing the serosurvey data to the modeled results as a possible method of validation; we agree that theoretically that would be an ideal approach. However, the only seroprevalence data in Ethiopia that is available is from 1994 and 1999 – both of which in the period before subnational case data were available. As additional data sources to compare model results may be collected or released, we are keen to continue to validate our model and related outputs. We have added a limitation to our discussion (lines 832-834) to note the challenges on validating this model to real-world trends that reads as:

“Additionally, in the absence of recent seroprevalence data or alternative data sources, it was challenging to validate the results our modelled results in Ethiopia to recent real-world trends.”

5. Discussion. More discussion on the limitations to practical use in this, or other, contexts e.g. data requirements, computing requirements? Are there other practical issues (e.g. other reporting artefacts) that might need to be addressed that didn’t come up for Ethiopia?

We thank the reviewer for the opportunity to discuss. Most of the practical limitations of this work are related to the computing requirements as well as the time and expertise needed to construct the model or adapt to new settings (with different data issues and epidemiological history), as most of the data used in this study (besides subnational case notification data are publicly available). We have added the following to our limitations section (lines 859-868) to discuss these considerations, including access to distributed computing environments for model experiments and increasing the number of subnational units, that now reads as:

“There are several practical limitations to our analysis as well, that could prevent this work from being expanded more broadly. We note several computational challenges that we were able to explore as we had access to substantial computational resources and a distributed computing environment. Our proposed model fitting solution yielded more reasonable computational times for fitting models in Ethiopia but still took approximately 5 hours to finish despite being run in a distributed computing environment; this approach may not be feasible in a local computing environment. Additionally, this model in Ethiopia only had 79 zones; many other countries, such as Nigeria or India, have 10 times or more second-administrative units. While this model is generalizable to other settings, practical considerations around resources to scale this model to other locations would need to be considered.”

**Minor comments**

6. Subnational scale. Is admin-2 small enough to identify subnational areas of susceptibility, and is this the same scale on which vaccination campaigns are organised?

We thank the reviewer for their question. While there are significant relative susceptibility gaps that emerge in Ethiopia at any subnational scale (i.e., first-administrative unit, second-administrative unit, etc.), further geographic resolution will also always show additional heterogeneity. However, we had access to case data and vaccination coverage estimates available at the second-administrative level (i.e., zones) and further geographic resolution would have required making substantial assumptions about the distribution of cases within a zone to third-administrative unit (i.e., woredas). Additionally, as the reviewer suggests, the second-administrative unit level is the scale for which subnational coverage monitoring targets are implemented and operationally it would be reasonable to organize a campaign.

7. Sporadic case reporting. The authors note “it was difficult to tell from the data whether the zones that reported alternating weeks with zero cases represent the total sum of cases across two weeks or whether these are missing other values that were not reported”. Can any of the co-authors in Ethiopia confirm this, and perhaps provide any explanation as to why this happens?

We (the Ethiopian co-authors) are unfortunately unable to identify the underlying mechanism for how weeks with zero cases would be reported and collated differently than weeks that had no reporting. Overall, we agree that this case reporting mechanism made interpreting the case data challenging.

8. Vaccine coverage estimates: can you clarify in the main text whether these estimates are based on administrative coverage, or coverage surveys?

We have modified our brief description of the vaccine coverage estimates in the main text to now also additionally include the following (lines 257-266), including data sources:

“In short, household-based survey data on MCV1 and MCV2 coverage are geolocated to the most geographically granular level possible. For RI MCV1 coverage, we used a model-based geostatistical framework to estimate coverage under the assumption that coverage is more similar in years and locations that have similar covariate patterns and in years, locations and age groups that are closer across time, space and age. For RI MCV2 coverage, less data was available to include in models, as MCV2 was only introduced in Ethiopia in 2019. Therefore, we used a hierarchical model at the zone level. For combined RI and SIA coverage, we developed a model tracking cohorts through time, accounting for RI coverage increases along with doses reported to be administered during SIAs.”

9. Model notation: α is used for both the amplitude in the definition of β_i, and in the definition of the force of infection (FOI).

We thank the reviewer for noting this confusing previous notation. We have updated our model notation for a handful of parameters for more clarity and consistency, including the notation for amplitude (which is now $A$).

10. Parameter values. Why have you chosen the overdispersion parameter φ = 5 in the definition of reported cases?

We tested various overdispersion parameters in initial model fitting experiments, across a wide range of values (i.e., 1, 5, 10, 50) to test if allowing additional variance or dispersion would improve acceptance of proposed samples in our samplers. We selected 5 as values greater than 5 did not improve likelihood acceptance. To add additional context for readers, we have added the following on lines 420-423:

“We tested various dispersion parameters (i.e., 1, 5, 10, 50) to improve likelihood acceptance in early modelling experiments. Increasing the dispersion parameter greater than 5 did not improve initial sampler acceptance rates, so therefore we used 5 for the remainder of experiments.”

Bibliography

1.	Sbarra AN, Rolfe S, Nguyen JQ, Earl L, Galles NC, Marks A, et al. Mapping routine measles vaccination in low- and middle-income countries. Nature. 2021 Jan;589(7842):415–9.

2.	Mosser JF, Gagne-Maynard W, Rao PC, Osgood-Zimmerman A, Fullman N, Graetz N, et al. Mapping diphtheria-pertussis-tetanus vaccine coverage in Africa, 2000–2016: a spatial and temporal modelling study. The Lancet. 2019 May 4;393(10183):1843–55.

3.	Prem K, Cook AR, Jit M. Projecting social contact matrices in 152 countries using contact surveys and demographic data. PLOS Comput Biol. 2017 Sep 12;13(9):e1005697.

4.	GBD 2019 Diseases and Injuries Collaborators. Global burden of 369 diseases and injuries in 204 countries and territories, 1990-2019: a systematic analysis for the Global Burden of Disease Study 2019. Lancet Lond Engl. 2020 Oct 17;396(10258):1204–22.

5.	Nuñez JR, Anderton CR, Renslow RS. Optimizing colormaps with consideration for color vision deficiency to enable accurate interpretation of scientific data. Malo J, editor. PLOS ONE. 2018 Aug 1;13(7):e0199239.

6.	Submission Guidelines [Internet]. [cited 2024 May 17]. Available from: https://journals.plos.org/plosone/s/submission-guidelines#loc-references

7.	Minta AA. Progress Toward Measles Elimination — Worldwide, 2000–2022. MMWR Morb Mortal Wkly Rep [Internet]. 2023 [cited 2024 May 26];72. Available from: https://www.cdc.gov/mmwr/volumes/72/wr/mm7246a3.htm
