# Supplementary material for: Fitting dynamic measles models to subnational case notification data from Ethiopia: Methodological challenges and key considerations
Source: PLoS Comput Biol. 2025 Apr 16;21(4):e1012922. doi: 10.1371/journal.pcbi.1012922 (PMC12002527; doi:10.1371/journal.pcbi.1012922)
Supplement: Attachment — Submitted filename: eth_case_fitting_rtr2_20241027_clean.docx [file pcbi.1012922.s005.docx]

Reviewer #1:

It is unclear to me why the authors would both suggest that the code repository is incorrect yet provide a README for a repository which is not the one used. I am unable to review the code but am otherwise satisfied with the responses given. Apologies for the short review but I am slightly pressed for time currently.

We thank the reviewer for their previous feedback and their acknowledgement of our previous responses.

We also appreciate the reviewer noting that, in order to show good faith effort in response to the reviewer’s original feedback, we added a README to the repository link currently available online. However, we are unable to finalize the code at this stage in a perpetual repository, since the work is still under review. The final version of the code, along with the added README file, will reside at this URL: https://github.com/ihmeuw/subnational_measles_ethiopia.

Reviewer #2:

This is a second review of the manuscript Exploring the utility of subnational case notifications in fitting dynamic measles models in Ethiopia. As stated in my initial review, this paper tackles the relevant and timely question of whether modeling efforts can be used to estimate subnational measles susceptibility in moderate burden areas, here Ethiopia.

In my initial review I commented that the paper lacked clear direction, seeming to be in between a methods paper on the challenges of building this type of model in data frail environments and a paper more focused on the direct results of the specific implementation for the Ethiopian context. I recommended that the Authors shift towards the first of these options, focusing on both the difficulties faced and the solutions that they were able to find (which were well thought out and clever). In this new version of the paper, the Authors have generally taken this advice, characterizing the work as a case study.

While this shift in focus is appreciated, the paper does not seem to have fully transitioned to a methodological focus. The results section remains oriented towards the specific outputs of the given implementation rather than a discussion of the methodological challenges themselves, which could have taken center stage were the paper to be more centralized around how to develop models in similar contexts.

We thank the review for their thoughtful feedback and appreciate their additional detailed review of our manuscript. We have responded to each specific consideration of theirs below, including reframing our methods and results section to account for their comments on shifting the focus to be methods focused.

**Points appreciated in the revision:**
In the methods section, the Authors have done a good job providing improved explanation of the model and its various aspects; for example explanation of the computational framework required, the choice of certain statistical parameters, and the choice of constant values.

We thank the reviewer for their positive feedback and ongoing support. Their suggestions strengthened our manuscript.

In particular, expanded explanation of specific computational requirements, programming language / package specifics / versions is greatly appreciated and provides a good model of best practice.

We also thank the reader for their suggestions that strengthened the integrity and transparency of our manuscript.

The Authors offer an expanded discussion on vaccine effectiveness, which is appreciated.
There is improved acknowledgment of computational cost for implementability of these forms of modeling approaches.

We appreciate the reviewers positive feedback on our manuscript improvements in response to their comments.

Once again, critical to highlight the laudable commitment to open source code, the inclusion of local authors, and the explicit acknowledgement of the importance of local experts (found in the discussion). This is an excellent example of necessary good practice for research in the public health sphere in LMICs.

We thank the reviewer for the ongoing support of our research process and team.

Unfortunately, following revision I have a **few points of outstanding concern:**
The title is a bit odd, intuitively suggesting that the Authors compare models using subnational case data to those that do not, which is not what is presented.

We thank the reviewer for their comment. We have revised our title to now read as: Fitting dynamic measles models to subnational case notification data from Ethiopia: methodological challenges and key considerations

As stated above, the paper still fails to take a truly methodological focus. Discussion of methodological challenges in modeling in data frail environments is an important and underrepresented topic and there is great potential for this paper to highlight that issue. To do so, however, I would like to see the methodological challenges and solutions take focus as a result unto themselves rather than remaining in the methods description.

We appreciate their reviewer’s recognition of this important topic and acknowledge that we did not adequately respond to their initial feedback. In order to address this and further shift our manuscript to have a more methodologic focus, we have moved large parts of the methods section describing how the methodological challenges were addressed, to the results:

- We have moved lines 331-334 in methods section to the Results section, and modified it to now read as: “We explored data-related considerations including sporadic case reporting, vaccine effectiveness, and variation in reporting rates. Additionally, we considered the implications of steep likelihood surfaces and collinear parameters before selecting a final model fitting algorithm.”
- We have moved lines 455-615 in previous methods subsections with the following headers to the results section: *Data considerations for model fitting: sporadic case reporting; Data considerations for model fitting: vaccine effectiveness; Data considerations for model fitting: variation in reporting rates; Statistical considerations for model fitting: steep likelihood surface; Statistical considerations for model fitting: collinear parameters; Final model fitting algorithm*

It remains unclear if the models described are intended for moderate burden or high burden settings, and the definition of high burden is unclear; this issue is primarily seen in the introduction.

We thank the reviewer for their feedback. These models, as they have been developed, are applicable in settings with moderate or high burden. However, operationally, findings these models suggest may have different applications and implications depending on underlying burden. For example, moderate burden settings may be interested in targeting of vaccination campaigns (e.g., subnational versus national, or age-specific efforts) and high burden settings may opt national campaigns or routine immunization strengthening efforts, but have other priorities as underlying burden changes (e.g., vitamin A supplementation to reduce mortality in outbreak settings). As the reviewer notes, we do not recommend these models for use in low burden or elimination settings, as we are not considering finer or social networks that may be driving remaining transmission. Even in some moderate burden settings, there may not be enough cases in many subnational regions for very granular model fitting.

In our introduction section, we chose to highlight the utility of these models to specifically guide targeted campaigns by age or space; however, we recognize that specifically distinguishing countries as high, moderate or low burden or coverage may be overly simplifying complex situations and possible use cases of our work. We highlight on lines 183-187 that Ethiopia, a setting with modest coverage, has substantial within country heterogeneity, which may suggest opportunity for impact with subnational campaign targeting. To reflect these complex settings more explicitly in our initial framing in the introduction, we have made the following revisions:

- Lines 107-112: “Targeted interventions, such as subnational SIAs or specific routine immunization system strengthening efforts by location, might be most operationally useful in countries with moderate vaccine coverage, or those with known subnational or age-group coverage heterogeneity, compared to approaches targeting by age or geography that may not be as relevant in countries with low overall coverage (where all areas or subgroups need coverage improvements via, for example, nationally-targeted SIAs).”
- Lines 117: REMOVED -- “A similar motivation in moderate coverage settings could also exist for implementing routine immunization strengthening efforts by location.”
- Lines 119-121: “However, along with other operational considerations such as resources and local factors, it would be ideal to understand who is susceptible within a population to inform targeted intervention approaches in any setting.”

In the introduction the Authors discuss important aspects of persistent susceptibility being attached to "pockets" and social networks but little discussion is given as to whether the model as proposed can truly identify this type of hyper heterogeneous susceptibility. A discussion of whether such dynamics can be captured as well as when and where complementary field based approaches to assess local heterogeneity in immunity could/would be needed would have been appreciated.

We thank the reviewer for noting this limitation of our analysis – we are able to estimate susceptibility at the zone (i.e., second-administrative) level, but not currently at finer geographic resolutions. We have modified the following sentences to be more accurate regarding our references of “pockets” of susceptibility or transmission:

- Lines 113-117: “Alternatively, high coverage settings with persistent transmission among communities without explicit geographic division (e.g., among specific social networks), might need different strategies, such as strengthening of health systems and health system provisions as well as tailored communication efforts and discussions of the risks and benefits of vaccination, to increase coverage beyond the use of geographically or age-targeted campaigns.”
- Lines 655-660: “Considering subnational measles dynamics and heterogeneity is critical for identifying areas that, along with other operational factors, may benefit from targeted interventions to address local (i.e., zone-level) susceptibility that are responsible for driving ongoing measles transmission.”
- Lines 737-739: “Additionally, there may be either geographic or social clustering of measles vaccine coverage so that there are large pockets of highly connected unvaccinated persons even within zones.”

Additionally, we have added the following (lines 814-819) to our discussion of limitations to more accurately describe to readers what geographic level our model is able to estimate:

“Our model estimates at the zone-level (i.e., second-administrative unit), which may not be able to accurately capture sub-zone level local transmission that may persist in hyper-localized or non-geographic social networks. Complementary field-based approaches, coupled with model types suitable for these data such as network or agent-based models, to assess local heterogeneity in immunity may be appropriate in settings with high coverage or communities experiencing challenges in immunization access or confidence.”

In the introduction and discussion, the Authors seem to claim that identification of subnational susceptibility is critical to effective targeted SIA policy. While identifying susceptible pockets is of course relevant, this statement feels perhaps too strong and they do not provide justification of why this type of approach is truly critical against simpler local solutions that look at coarser grained estimates of low vaccine coverage. It also ignores other aspects that go into targeted immunization campaigning, such as consideration of populations most at risk, resource constraints, and other local factors.

We thank the reviewer for the comment and the ability to modify our language. We agree with the reviewer that there is more to consider than subnational coverage or susceptibility and that results from the type of model that we have developed need to be weighed along with other operational considerations; we have previously noted this on lines 140-143. As the reviewer notes, consideration of populations most at risk – instead of estimates of coverage alone – are one utility of using modeling approaches to estimate susceptibility across age and space. We highlight this on lines 121-125. This of course is limited to being able to accurately capture the most vulnerable populations (e.g., internally-displaced persons), who are often not captured in coverage or case data.

To more accurately describe the contributions of susceptibility estimates in targeting campaigns, however, we have modified the following in our introduction section:

- Lines 105-107: “Recently, to contribute to informing these operations, there have been increased efforts to map geographic variation in vaccination coverage across low- and middle-income countries (LMICs), including Ethiopia.”
- Lines 119-121: “However, along with other operational considerations such as resources and local factors, it would be ideal to understand who is susceptible within a population to inform targeted intervention approaches in any setting.”
- Lines 123-125: “However, informing policy decisions based on vaccination coverage estimates alone would provide an incomplete picture in places where there are large amounts of natural immunity.”
- Lines 143-147: “To contribute to these decisions, instead of relying on the coverage estimates alone, mathematical models incorporating transmission dynamics can potentially be used to incorporate not only information on vaccine coverage, but also demography, contact patterns, person mobility, and case notifications, to estimate the dynamics of measles within a community and quantify measles susceptibility.”

Additionally, we have modified the following in our discussion section:

- Lines 655-660: “Considering subnational measles dynamics and heterogeneity is critical for identifying areas that, along with other operational factors, may benefit from targeted interventions to address local pockets of susceptibility that are responsible for driving ongoing measles transmission.”
- Lines 843-844: “Understanding subnational susceptibility is essential to contribute towards operational decisions for planning targeted interventions.”

Particularly in the introduction the Authors conflate vaccine effectiveness with efficacy, which should be corrected.

We apologize for our error. Reference to “vaccine efficacy” (line 164) has been revised to read as “vaccine effectiveness”. Additionally, in the discussion (line 690), we have revised our reference of “efficacy” to “effectiveness”.

While the addition of acknowledging rubella misclassification is appreciated, this discussion is underdeveloped and the generalizability of the same model across settings of differential rubella burden is not justified. This remains particularly important in areas that continue to use mono antigen measles vaccine, which is true of several high burden settings.

We thank the reviewer for the opportunity to further expand on our discussion of rubella misclassification. In our previous draft, we largely failed to discuss the operational complexity regarding laboratory-based or rapid diagnostics for measles IgM confirmatory testing. In many settings not yet approaching elimination, which are those that also still experience rubella burden, the proportion of suspected cases that have IgM confirmation is relatively low, however quite variable. We have modified our discussion on rubella misclassification to now read as follows (lines 772-782):

“We only considered suspected measles cases and did not consider lab-confirmed measles cases or adjust for test positivity rates. Therefore, the cases we considered in this model may have been misclassified as measles instead of, for example, rubella. As rapid diagnostic tests for measles and rubella are still under development and programmatic evaluation, many locations, including those that experience simultaneous measles and rubella burden, do not currently have access to adequate confirmatory testing for suspected measles cases. Therefore, in order to mitigate rubella misclassification, approaches that only consider measles IgM positive cases may miss a substantial proportion of true measles cases. More exploration is necessary to examine whether there is any additional information that can be learned about reporting rates and possibly increased usability of case notifications once rapid diagnostics become available for widescale use.”

In my prior review I mentioned the need for more clarity on operational utility and model validation. While within the context of shifting the focus to methodological challenges specific operational recommendations for Ethiopia become less pertinent, the broader need for a discussion of validation and operational purpose remains relevant. It is important for the Authors to critically consider both how such a model can/should be used and whether it is justifiable for countries to be asked to buy into a model that would likely create not only financial burden but a dependence on (likely) global north countries for continued implementation. While the Authors note that this tool should be used not as a replacement for but rather in concert with simpler local tools, a deeper reflection on why such addition is necessary/warranted would have been greatly appreciated. It is worth noting here that a perfectly acceptable explanation is that such modeling efforts could be justified as "one off" validations assessing how well (or not) simpler methods work compared to more statistically complex approaches; but such interpretation should be made explicit.

We appreciate the reviewer’s reflection on the added benefit of complex modeling approaches especially with respect to the current reliance on resources from the global north. One explanation is as the reviewer suggests -- this modeling exercise was needed to assess whether more complex methods provide additional benefit over simpler local approaches. However, this modeling exercise also, for the first time, explicitly estimated subnational case ascertainment, which local approaches, as they currently exist, cannot do. We note the importance of considering these subnational case ascertainment rates in our discussion; however, to better reflect these motivations, we have modified the following sentence (lines 667-669) in the beginning of our discussion to read as :

“We explored the utility of using case notifications in subnational transmission models and validity of modelling approaches to estimate susceptibility and case ascertainment. We faced challenges related to data quality and statistical and computational complexity.”

Additionally, we describe practical limitations of this work being adapted in local contexts in the global South on lines 826-841 and have made the following modifications so it now reads as:

“There are several practical limitations to our analysis as well, that could prevent this work from being expanded more broadly and attempted in local contexts. We note several computational challenges that we were able to explore as we had access to substantial computational resources and a distributed computing environment. Our proposed model fitting solution yielded more reasonable computational times for fitting models in Ethiopia but still took approximately 5 hours to finish despite being run in a distributed computing environment; this approach may not be feasible in a local computing environment. Additionally, this model in Ethiopia only had 79 zones; many other countries, such as Nigeria or India, have 10 times or more second-administrative units. While this model is generalizable to other settings, practical considerations around resources to scale this model to other locations would need to be considered. Additionally, we acknowledge the current scope of resources (e.g., time, expertise) in many high burden settings and the subsequent challenges that adapting these methods in these local contexts. Given these limitations broadly along with ongoing operational practices (i.e., making prioritization decisions based on triangulating estimates of vaccination coverage), modelled results of this kind currently are best suited to be used alongside existing processes and data streams as an additional resource for decision-makers.”

In particular, a more thoughtful discussion of how such a model could be validated, and the limitations of such validation remains critical. Ethically, if such models are meant to be operationalized for decision making, it is necessary to have a mechanism of validation. At present, the Authors do not fully address this issue. In the context of a methods based paper, this could be approached from a perspective of challenges in such validation; but it should still be meaningfully considered. If the conclusion is that such validation may be unrealistic in the context of application, a discussion of the ramifications of that conclusion is warranted.

We agree, as the reviewer notes, that validating these models in the context of methods development and data fragility can be challenging. Additionally, we required vaccination coverage data as input data for fitting and would thus be inappropriate to validate against that. Also, seroprevalence surveys were only available for years prior to when we had case data and could actually fit the model, also limiting our ability to validate against these sources. Given already limitations associated with the case data available that we tried to address in our modeling framework, it would be challenging to use an additional year of case data not adjusted for case ascertainment as our comparison. We tried as best as possible to validate our model against outbreak reports from the available published and grey literature (lines 744-762); however, as we note on lines 754-761, this also is challenging as our model describes a pre-COVID pandemic world and these outbreaks reflect a post-COVID and post-war/conflict/famine landscape. Finally to note, the objectives of this exercise outlined (i.e., understanding the utility of subnational modelling, identify data limitations, overcome technical challenges, involve local stakeholders in discussions) do not involve direct input into policy making; however, this exercise does not have to directly inform policy to still be useful.

To discuss this further, we have added the following to our discussion (lines 762-768):

“Models, including those presented in this work, would require validation prior to their use to guide operational decisions. However, these validations remain challenging in light of evolving epidemiologic and socio-political circumstances and in the absence of gold-standard data to validate models against, including a lack of available recent seroprevalence data. Identifying opportunities for enhanced model validation will be critical, though, for the uptake of these frameworks for decision-making.”

I would like to underscore that despite the concerns sighted above, modeling work like this remains deeply relevant and, in particular, work like this that clearly describes the methodological difficulties and potential solutions that could be used in those efforts is a valuable contribution. These types of models are a point of active research and the absence of publications discussing more technical points and challenges of implementation only acts to limit the progress of the field.

We thank the reviewer for this feedback and appreciate their acknowledgement of the importance of our work and valuable contributions of this manuscript.
